# Paired Rheumatoid Arthritis Synovial Biopsies From Small and Large Joints Show Similar Global Transcriptomic Patterns With Enrichment of Private Specificity *TCRB* and TCR Signaling Pathways

**DOI:** 10.3389/fimmu.2020.593083

**Published:** 2020-11-23

**Authors:** Clement Triaille, Louise Vansteenkiste, Manuel Constant, Jérôme Ambroise, Laurent Méric de Bellefon, Adrien Nzeusseu Toukap, Tatiana Sokolova, Christine Galant, Pierre Coulie, Javier Carrasco, Patrick Durez, Bernard R. Lauwerys

**Affiliations:** ^1^Pôle de Pathologies Rhumatismales et Systémiques, Institut de Recherche Expérimentale et Clinique, Université catholique de Louvain, Brussels, Belgium; ^2^Department of Pediatric Haematology and Oncology, Cliniques Universitaires Saint-Luc, Brussels, Belgium; ^3^Laboratory of Translational Oncology, Institute of Pathology and Genetics/Grand Hôpital de Charleroi, Gosselies, Belgium; ^4^Centre de Technologies Moléculaires Appliquées, Institut de Recherche Expérimentale et Clinique, Université catholique de Louvain, Brussels, Belgium; ^5^Department of Rheumatology, Cliniques Universitaires Saint-Luc, Brussels, Belgium; ^6^Department of Pathology, Cliniques Universitaires Saint-Luc, Brussels, Belgium; ^7^de Duve Institute, Université catholique de Louvain, Brussels, Belgium

**Keywords:** rheumatoid arthritis, synovial biopsy, gene expression, T lymphocytes, TCR repertoire

## Abstract

**Objectives:**

We explored histological and transcriptomic profiles of paired synovial biopsies from rheumatoid arthritis (RA) patients, in order to assess homogeneity in synovial tissue at the individual level.

**Methods:**

Synovial biopsies were performed simultaneously in one small and one large joint per patient using needle-arthroscopy for the knee and ultrasound-guided biopsy for the hand or wrist. Synovium from individuals with osteoarthritis was used as controls. Paraffin-embedded samples were stained for CD3, CD20, and CD68. Total RNA was hybridized on high-density microarrays. *TCRB* variable sequences were obtained from synovial and blood RNA samples.

**Results:**

Twenty paired biopsies from 10 RA patients with active disease were analyzed. Semi-quantification of histological markers showed a positive correlation for synovial hyperplasia, inflammatory infiltrates and CD3-positive T cells between pairs. Pairwise comparison of transcriptomic profiles showed similar expression of RA-related molecular pathways (TCR signaling, T cell costimulation and response to TNFα). T cells clonotypes were enriched in all but one joints compared to blood, regardless of the magnitude of T cell infiltration. Enriched clonotypes were shared between pairs (23–100%), but this was less the case in pairs of joints displaying weaker T cell signatures and more pronounced germinal center-like transcriptomic profiles.

**Conclusion:**

Cellular and molecular alterations in RA synovitis are similar between small and large joints from the same patient. Interindividual differences in magnitude of T cell infiltrates and distribution of enriched T cell clonotypes support the concept of distinct synovial pathotypes in RA that are associated with systemic versus local antigen-driven activation of T cells.

## Introduction

Synovitis in rheumatoid arthritis (RA) is characterized by alterations in synovial architecture such as hyperplasia of the synovial lining membrane, hypervascularity and infiltration of the sublining by mononuclear cells. These modifications are associated with changes in the activation patterns of infiltrating and resident cells, as indicated by recent molecular studies on whole biopsies or single synovial cells (1–4).

Several studies were performed in order to understand the mechanisms driving heterogeneity in histological, cellular and molecular changes in rheumatoid synovitis and investigate whether synovial cellular and molecular patterns can be linked to clinical outcomes (5–11). Thus, clinical variables such as disease activity, disease duration, ACPA status or response to specific therapies were associated with variations in cellular populations and molecular profiles in RA synovial biopsies (12). We and others reported that disease activity correlates with the expression of T cell- and myeloid-related transcripts in RA synovitis (2, 6, 11). TNF blockade results in a preferential decrease in the proportion of CD68-positive macrophages and myeloid-associated transcripts (5) while tocilizumab rather impacts CD3-positive T cells and T cell-related molecular functions (13). In addition to the effects of extrinsic factors, evidence also indicates that intrinsic variations in synovial architecture determine the organization of RA synovitis. Synovial pathotypes (lympho-myeloid, myeloid or pauci-immune) were defined based on the relative enrichment of cell populations and gene expression profiles (9, 10). The stability of these pathotypes over time, and whether they reflect the presence of distinct endotypes (i.e. subgroups defined by different functional mechanisms) or extremities of a continuous spectrum, is still under investigation.

Most studies in the field were performed on synovial samples regardless of the joints they were harvested from. Nevertheless, the possibility that joint-to-joint variations (especially large versus small joints) contribute to the heterogeneity of RA synovitis needs to be considered. In 2002, Kraan et al. compared cell numbers in synovial biopsies from large and small joints harvested in 9 RA patients and found significant correlations of CD3+ T cells, CD68+ macrophages and CD138+ plasma cells across pairs of joints (14). More recently, Musters et al. compared TCRβ repertoires in 9 pairs of RA ankle or knee joints and reported a range of 20 to 60% overlap between the 25 most frequent clones in each pair of joints (15).

Global molecular profiling technologies are being evaluated in diagnostic and theranostic developments in the field of synovial biopsies. Hence, the present study intended to evaluate whether high-throughput expression studies, combined with massive *TCRB* sequencing and conventional histology/immunohistochemistry assessments, delivers similar signals across pairs of joints from the same patient. Our results demonstrated that global molecular signals are similar in two different joints from the same patient. *TCRB* sequences that are enriched in synovial tissue compared to blood are also shared between pairs of joints. However, this is less frequently the case in synovial biopsies with weak T cell infiltration, in which transcriptomic profiling points at preferential T cell organization in germinal centers.

## Materials and Methods

### Patients and Samples

Ten patients with active RA were included in the study. All patients met the ACR/EULAR 2010 RA classification criteria (16). Demographic and clinical characteristics of the patients are displayed in **Table 1**. The study was approved by the ethics committee of the Université catholique de Louvain. All patients gave written inform consent to participate in the study.

**Table 1:** Demographic and clinical characteristics of the RA patients (n = 10) included in the study.

Age (mean ± SEM)	51.7 ± 4.4 years
Disease duration (years) (mean ± SEM)	12.1 ± 3.6 years
Female	10/10
ACPA/RF positivity	8/10
Erosive disease	10/10
Ongoing treatment	
cDMARDS	4/10
bDMARDS	4/10
Prednisolone (<7.5mg/d)	4/10
No DMARDS	2/10
DAS28CRP (mean ± SEM)	5.07 ± 0.28
VAS physician (mean ± SEM)	5.07 ± 0.41
CDAI (mean ± SEM)	27.48 ± 2.4
SDAI mean ± SEM)	29.79 ± 2.7
CRP (mg/l) (mean ± SEM)	23.44 ± 7.54
HAQ (mean ± SEM)	1.73 ± 0.24

ACPA, anti-citrullinated protein antibodies; bDMARDS, biological disease-modifying anti-rheumatic drugs; CDAI, clinical Disease Activity Index; cDMARDS, classical disease-modifying anti-rheumatic drugs; CRP, C-reactive protein; DAS28CRP, disease activity score-28 for rheumatoid arthritis with CRP; HAQ, Health Assessment Questionnaire; RF, rheumatoid factor; SDAI, simplified Disease Activity Index; VAS, visual analog scale.

For each patient, synovial biopsies were obtained the same day from a swollen small (metacarpo-phalangeal joint or wrist) and a large (knee) joint, using a US-guided biopsy procedure in small joints and needle-arthroscopy in large joints. Six to 10 synovial biopsy fragments were obtained from each joint. Half of them were stored at −80°C after overnight incubation in RNA-Later solution (Invitrogen). The other half was fixed overnight in 10% formalin buffer at pH 7.0 and embedded in paraffin for histologic and immunohistochemistry analyses. Whole blood from each patient was collected in PAXgene Blood RNA tubes (BD Biosciences) and stored at −20°C. Synovial biopsies from the knee of 4 osteoarthritis (OA) patients were used as control for the transcriptomic studies.

### High-Density Transcriptomic Studies

Total RNA was extracted from the synovial biopsies using a NucleoSpin RNA II extraction kit (Macherey-Nagel), including DNase treatment of the samples. RNA quality was assessed using an Agilent 2100 Bioanalyzer and RNA nanochips. Complementary RNA (cRNA) was synthesized from 100 ng total RNA, and biotin-labelled according to a standard Affymetrix procedure (GeneChip 3′ IVT Plus). GeneChip Human Genome U133 Plus 2.0 arrays were hybridized overnight at 45°C with 10 μg fragmented biotinylated cRNA. The slides were then washed and stained using a EukGE‐WS2v5 fluidics protocol on a GeneChip Fluidics Station 450, before being scanned on a GeneChip Scanner 3000 (Affymetrix). The Affymetrix.CEL files were deposited in the Gene Expression Omnibus of the National Center for Biotechnology Information, and are accessible through Gene Expression Omnibus accession number GSE153015.

### Histologic and Immunohistochemical Analyses

Serial histologic sections were stained with hematoxylin and eosin. The following parameters were evaluated: synovial hyperplasia, lymphoplasmacytic cell infiltrates, fibrinoid necrosis and vascular hyperplasia. Immunolabeling experiments were performed using a standard protocol, as previously described (13). The following antibodies were used: anti‐CD3 (Neomarkers), anti‐CD20 (Biocare Medical), anti‐CD68 (DakoCytomation). Evaluation of all histological and immunohistochemical variables was performed independently for each joint by an expert anatomopathologist blind to clinical data, using a semiquantitative score on a 0 to 3 scale, where 0 indicates absence of the feature and 3 represents the highest level. A specific score was assigned for the extent of hyperplasia of the synovial lining layer, where 0 indicates one or two affected cell layers, 1 indicates three or four affected cell layers, 2 indicates five or six affected cell layers, and 3 indicates at least seven affected cell layers.

### *TCRB* Transcripts Sequencing

Total RNA was extracted from whole blood samples using Maxwell RSC simplyRNA blood kits (Promega) on a Maxwell RSC Instrument (Promega). Total RNA (250 ng for synovial samples and 250 to 1,088 ng for blood samples) was engaged in a reverse transcription reaction using Superscript III reverse transcriptase (Invitrogen) and primers specific for *TCRB* CDR3 regions and housekeeping gene *RPP30*. For quality control purposes, the amount of *TCRB* cDNA was quantified by qPCR before the construction of *TCRB* libraries. In addition, this quantification step, together with *RPP30* normalization, allowed to compare global *TCRB* expression between joints. During the reverse transcription step, each *TCRB* cDNA molecules was tagged with a unique genetic barcode (Unique Molecular Identifier, UMI). Libraries of *TCRB* rearranged sequences were built from the obtained cDNA by targeted amplification. After addition of Illumina required adaptors, libraries were sequenced in a MiSeq platform (Illumina). Reads were aligned using MiXCR to identify individual *TCRB* rearrangements. UMIs were used to compile reads derived from each initial cDNA molecule, thereby correcting amplification biases or sequencing errors introduced during the library construction or sequencing process, respectively (17). UMIs also provided an absolute quantification of sequenced *TCRB* sequences. The latter were grouped by unique productive rearrangements, identifying T cell clonotypes. To identify the clonotypes that were enriched in synovium compared to blood (Enriched T cell Clonotypes, ETC), we compared the frequency of each clonotype in the joint repertoire to that in a random sub-sample of 20,000 *TCRB* sequences from blood molecules. If a clonotype was not detected in this sub-sample, its estimated blood frequency was considered as ≤ 1/20,000. Finally, an ETC was defined as follows: (1) the clonotype must be represented by at least 10 *TCRB* molecules in the synovium and (2) its frequency in synovium must be ≥ 100-fold the frequency in blood (p-value < 1 × 10^−6^). ETC were considered as shared between joints when the same *TCRB* rearrangement was detected in the other joint of the patient. All *TRCB* sequencing procedures were run in duplicates (**Supplementary Figure 1**), showing that reproducible results were obtained for clonotype frequencies > 0.5% (which was the case for all ETC).

### Statistical Analyses

Analyses of the microarray data were performed using GeneSpring GX software (Agilent). Fluorescence intensity data were normalized using robust multiarray analysis. RA versus OA differences in gene expression were assessed using moderated *t*‐tests. RA small joint versus large joint differences in gene expression were evaluated using moderated paired *t*-tests. Pathway‐enrichment analyses were performed using GeneSpring GX (Gene Ontology Annotations). Gene scores were used in some analyses to assess the overexpression of transcripts belonging to the same pathways. They were calculated as the median value of the fold changes of each transcript belonging to the pathway in a given RA sample compared to its average expression value in the OA samples. Distribution of synovial biopsies in T cell-poor versus T cell-rich samples was guided by the results of a supervised hierarchical clustering study using transcripts belonging to the TCR signaling Gene Ontology pathway.

Intra-class Correlation Coefficients (ICC) were calculated using the irr R package. For each probe, a one-way ICC was computed to assess the variation of expression of the gene within each real pair of samples compared to the variation in all samples. The same process was applied using random pairs compared to the variations in all samples. All other descriptive statistics used in this report were calculated using Prism 5 (GraphPad).

## Results

**Figure 1:**
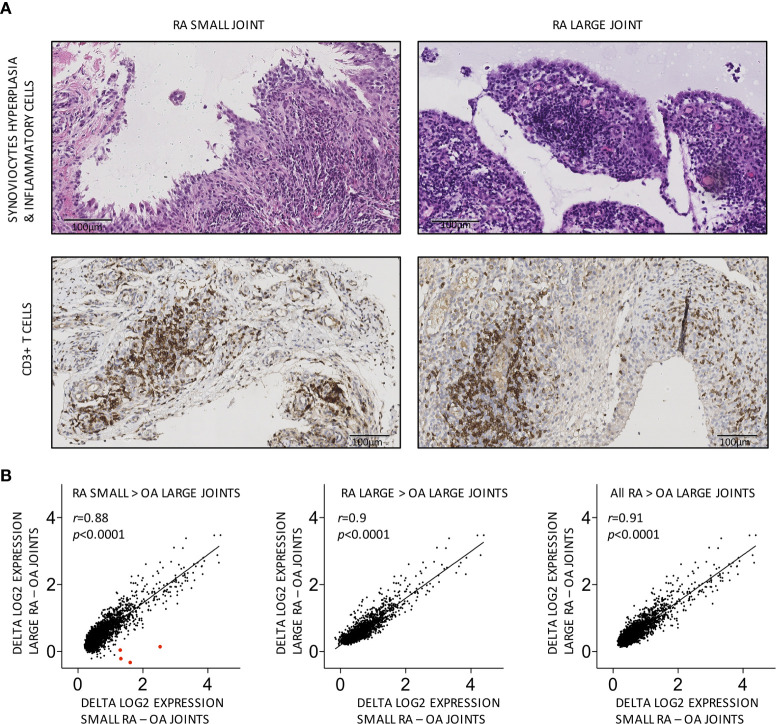
Similar cellular and global molecular characteristics in small versus large joints from the same RA patients. **(A)** Representative pairs of biopsy sections obtained from small versus large RA joints from the same patient, showing similar grades of synovial hyperplasia and CD3+ T cell infiltration. **(B)** Transcripts overexpressed in small, large or all (small and large) RA joints display similar patterns of differential expression in small versus large RA compared to OA joints. Each dot represents the difference in log2-transformed gene expression values in small RA (X axis) or large RA (Y axis) minus OA joints. Transcripts encoding HOXD10 and HOXD11 are shown as large red dots in the first panel. Pearson *r* correlation coefficients and *p* values are displayed on the graph.

We first performed semi-quantitative evaluations of conventional histological scores (synovial hyperplasia, inflammatory infiltrates, vascularity, fibrinoid necrosis) and CD3, CD20 and CD68 positive cells in paired synovial biopsies obtained from large (needle-arthroscopy) and small (US-guided biopsies) swollen joints from 10 patients with active RA. As shown in **Table 2** (and **Figure 1A**), significant correlations were found for synovial hyperplasia, inflammatory infiltrates and CD3+ T cells between pairs of samples.

**Table 2:** Comparison of semi-quantitative evaluations of histological and immunohistochemistry scores in paired synovial biopsy sections obtained from small versus large joints of 10 RA patients.

	Median score in small joints (interquartile range)	Median score in large joints (interquartile range)	Spearman *r* correlation coefficient	Correlation *p* value
Synovial hyperplasia	1 (1–2)	2 (0.75–2)	0.7473	0.0174
Inflammatory infiltrates	2 (1–2)	2 (0–2)	0.6158	0.0478
Hypervascularity	1 (1–2)	2 (1–3)	0.2888	0.4069
Fibrinoid necrosis	0 (0–1)	1 (0–2)	−0.276	0.4023
CD3+ cells	1.5 (1–1.5)	1.5 (0.5–2)	0.7936	0.0047
CD20+ cells	0.5 (0–1)	0 (0,1–5)	0.2308	0.4854
CD68+ cells	1 (1–2)	2 (0.5–2)	0.5884	0.0609

Next, we performed comparisons of high-throughput gene expression profiles between pairs of RA synovial samples. HGU133 Plus2.0 data sets were generated using large (needle-arthroscopic) versus small (US-guided) RA synovial biopsies and 4 osteoarthritis disease controls. 2,842, 1,995, and 3,303 transcripts were overexpressed in respectively all (n = 20), large (n = 10), and small (n = 10) RA synovial biopsies compared to OA controls. As shown in **Supplementary Figure 2**, these transcripts strongly overlapped. Most transcripts overexpressed in small RA joints compared to OA controls were equally overexpressed in large RA joints and vice versa (**Figure 1B**). Several HOX transcripts, known to be overexpressed in synoviocytes from small joints compared to large joints, were found accordingly overexpressed in small but not in large RA joints compared to OA controls (control OA synovial tissue was obtained from large joints).

**Figure 2:**
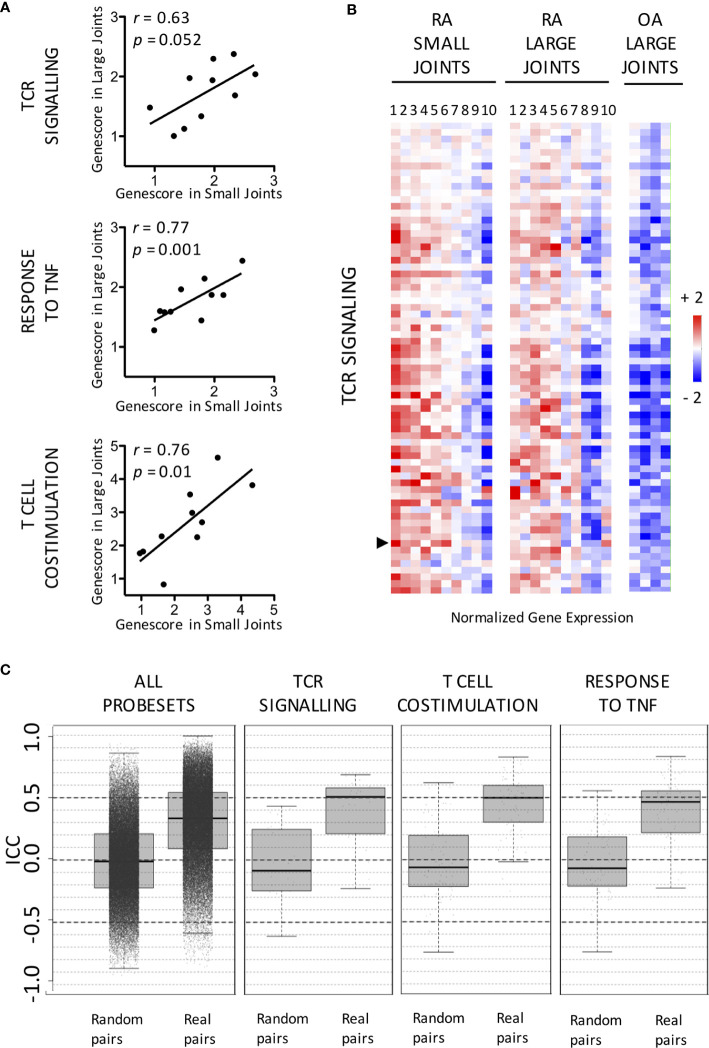
Similar expression patterns of RA-specific molecular pathways in small versus large joints from the same RA patients. **(A)** Distribution in small versus large RA joints of gene scores calculated for the following pathways: TCR signaling, Response to TNF and T Cell co-stimulation. Pearson *r* correlation coefficients and *p* values are displayed on the graph. **(B)** Expression of TCR Signaling-related transcripts in paired small versus large RA joints compared to OA controls. Data are displayed as mean-centered log2-transformed expression values according to the color scale shown on the figure. The list of transcripts is available in **Supplementary Table 2**. PLCγ2 (indicated by an arrow) is differentially expressed between small and large joints using paired t-tests. **(C)** Distribution of Intra-Class Correlation Coefficients calculated for all transcripts involved in the following pathways TCR signaling, Response to TNF and T Cell co-stimulation, in real compared to random pairs of joints. Data are displayed as median values and interquartile ranges.

Not surprisingly, (gene ontology) pathway analyses indicated that transcripts overexpressed in RA samples were significantly enriched in numerous immune-related pathways (**Supplementary Table 1**). We further focused on genes involved in pathways known to play a critical role in RA pathogenesis: TCR-signaling pathway, T cell co-stimulation and cellular response to TNF (**Supplementary Table 2**). Although we observed a strong inter-patient variability in the synovial expression of these pathways, expression patterns in pairs of joints from the same patients were similar (**Figures 2A, B**). One transcript (CASP8) in the TNF response pathway and another one in the TCR signaling pathway (PLCγ2) (none in the T cell co stimulation pathway) were differentially expressed between large and small RA joints, but these differences were not significant when corrections for multiple comparisons were applied. Intra-class correlation coefficients were also calculated in order to compare intra- versus inter-patient variations in gene expression values. Using either all transcripts, or transcripts belonging to RA-specific pathways, median intra-class correlation coefficients were significantly higher in real (from the same individuals) compared to random pairs of samples, thereby confirming the similarity of gene expression patterns in pairs of joints from the same patients (**Figure 2C**).

We found strong correlations between all metrics of T cell infiltration in the synovium: semi-quantitative evaluation of CD3 positive cells by immunohistochemistry, evaluation of *TCRB* gene expression by qPCR, or computation of a TCR-signaling pathway gene score using microarray data (**Supplementary Table 3**). Thus, samples with high TCR signature had higher numbers of CD3 positive cells at immunohistochemistry and higher *TCRB* mRNA expression. We obtained *TCRB* CDR3 repertoires using total RNA from synovial biopsies and peripheral blood of RA patients and OA controls. We defined enriched T cell clonotypes (ETC) as clonotypes at least 100× more frequent in synovium compared to blood. We found ETC in 19 out of 20 synovial samples (**Supplementary Table 4**). Average number of ETC was 5.2 ± 0.9 per sample, regardless of the level of T cell infiltration in the tissue (low T cell signature: 6.25 ± 1.9; high T cell signature: 4.5 ± 0.8, *p* = 0.354). Joint location was not associated with number of detected ETC (small joints: 4 ± 2.36, large joints: 6.4 ± 5.04, *p* = 0.19).

**Figure 3:**
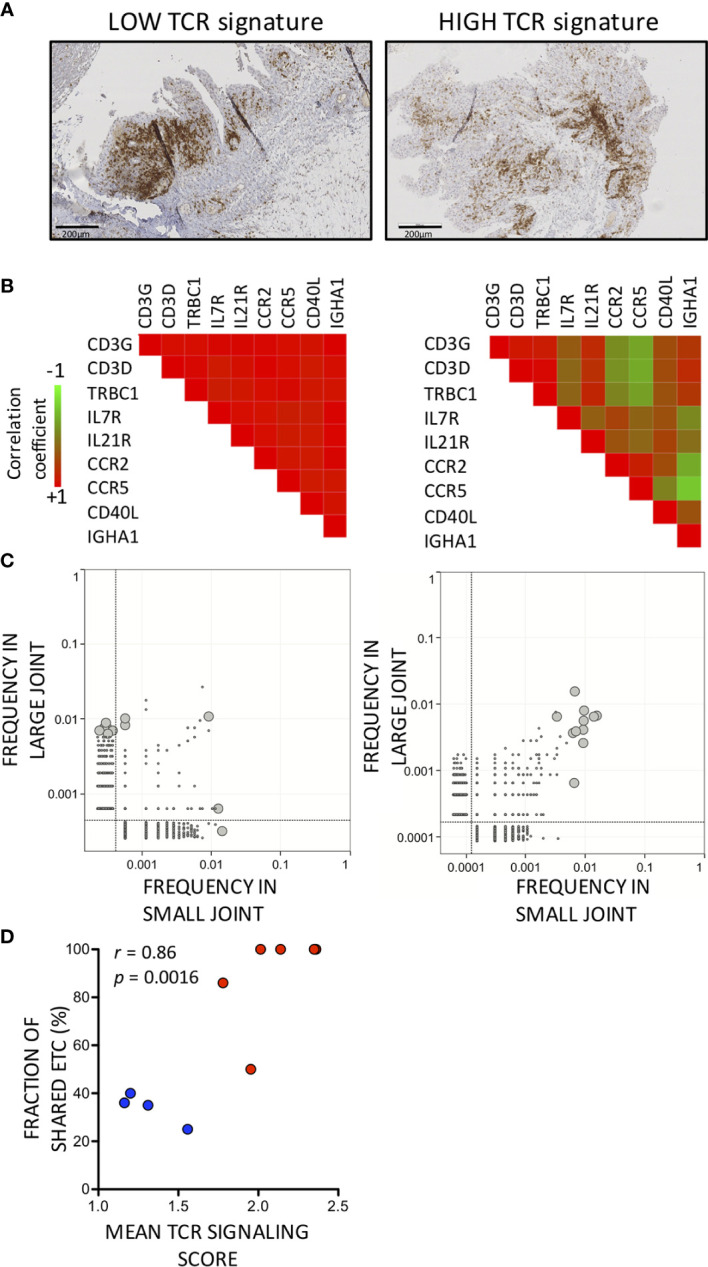
Enriched synovial *TCRB* sequences are shared more frequently between pairs of synovial biopsies with high compared to low T cell signatures. **(A)** Representative synovial biopsy sections illustrating differences in T cell disposition between low (left panel) versus high (right panel) T cell infiltration. CD3-positive cells are stained in brown. **(B)** Correlation coefficients between levels of expression of T cell-related transcripts and transcripts associated with germinal centers in synovial biopsies with a low (left panel) versus high (right panel) T cell infiltration. Pearson *r* correlation coefficients are displayed according to a color scale shown in the figure. **(C)** Enriched T cell Clonotypes are shared more frequently in T cell-rich compared to T cell-low synovial biopsy pairs. Two examples are displayed showing the distribution of *TCRB* sequences in a pair of T cell-low (left panel) and T cell high (right) panel synovial biopsies. The grey lines are the limit of detection. ETC (i.e. *TCRB* sequences 100x more frequent in a large or small synovial biopsy specimen compared to blood) are depicted as large gray circles. **(D)** Correlation between TCR signaling gene score (mean value of paired samples) and fraction of ETC shared between pairs of synovial samples. Blue dots indicate low while red dots indicate high TCR signaling scores.

Range of ETC shared between synovial biopsies from the same patients was 23% to 100%. We wondered whether variations in percentages of shared ETC were associated with differences in synovial characteristics and found that proportions of ETC shared between pairs of joints were higher in joints with a pronounced T cell infiltration. Thus, we found a significant correlation between fractions of shared ETC between pairs of samples and synovial levels of expression of T cell related transcripts (**Figures 3C, D**) or synovial CD3+ T cell infiltration at immunohistochemistry (data not shown), while absolute numbers of ETC were not influenced by the amplitude of T cell infiltration. In synovial samples having low levels of expression of T cell related transcripts, hence low fractions of ETC shared across pairs of samples, expression of T cell specific transcripts (CD3 or constant region of TCRβ chain) strongly correlated with transcripts encoding immunoglobulins, IL21R or IL7R, pointing at the presence of germinal centers in these samples (**Figures 3A, B**).

## Discussion

We compared global molecular profiles in small versus large synovial biopsies harvested simultaneously in 10 RA patients with active disease. We found that transcriptional signals were similar across pairs of synovial samples. Study of the TCRβ chains repertoire indicated that 19/20 synovial samples contained enriched (>100x) T cell clonotypes compared to blood. Enriched T cell clonotypes were shared between paired of joints with molecular and histological characteristics of pronounced T (and other immune) cell infiltration, but this was less the case in pairs of joints with a low inflammatory profile. In these joints, transcriptomic signals suggested that T cells preferentially organized in germinal-center like structures.

Evidence indicates that molecular profiling of synovial tissue in RA delivers valuable information on clinically relevant variables such as disease severity and response to therapy (2, 5–7, 9, 10). Whether these observations will improve decisions in daily clinical practice is the subject of ongoing large-scale prospective studies. In this process, it is of importance to identify potential sources of confusion, i.e., variations in molecular signals that are unrelated to clinically relevant outcomes. Among these, joint location is a definite candidate. Obviously, one expects to find differences in molecular and functional characteristics of resident cells in small compared to large joints. Frank-Bertoncelj et al. reported that the expression of coding, but also long non-coding and microRNAs located in the HOX loci was regulated by joint-specific epigenetic mechanisms (18). Accordingly, we did detect differences in the expression of HOX transcripts in small versus large joints. However, in the context of RA, inflammation-associated changes overwhelm normal joint-to-joint variations in gene expression signals. Thus, we found that pairs of joints from the same RA patients displayed concordant global molecular changes at the tissue level. By showing that joint location does not impact global transcriptomic signatures, these results are of importance for the clinical development of tests based on the detection of molecular patterns in RA synovial biopsies.

Noteworthy, our study did point at possible joint specific mechanisms of T cell activation, based on the comparative analysis of *TCRB* sequences distribution. In pairs of joints with low degrees of T cell infiltration, CD3 or TCR gene expression displayed very high levels of correlation with Ig, IL21R or IL7R gene expression, as is expected in actual germinal centers (19, 20). Several *TCRB* sequences were enriched in these biopsies, however, they were infrequently shared within pairs of samples from the same patients, which points at the possibility of local, joint-specific antigen-driven mechanisms of T cell activation. Importantly, lack of detection of shared *TCRB* sequences in these pairs of joints with low levels of T cell infiltration was not a matter of sensitivity, since these analyses were restricted to highly enriched clones, for which the representation in terms of numbers of transcripts was sufficient. As reported in previous studies, nodular germinal center-like aggregates were also present, even at a high frequency, in pairs of joints with higher amounts of T cells (21, 22), but they were surrounded by diffuse T cell infiltrates, thereby diluting the germinal center-associated transcriptomic signals found in these biopsies. In these biopsies, *TCRB* sequences enriched in synovial tissue were shared across pairs, which might point at a systemic antigen-driven T cell response.

Overall, our results give strong support to the hypothesis that synovial pathotypes are the manifestations of distinct mechanisms of inflammation in rheumatoid arthritis (local versus systemic) and are persistent across joints from the same patients. Whether these pathotypes translate into distinct clinical phenotypes in terms of disease progression or response to therapy is under intense scrutiny. The presence of ectopic lymphoid structures in RA synovitis is associated with the activation of immunologically relevant mechanisms: expansion of PD-1^hi^ CXCR5- “peripheral helper” CD4 T cells (23), production of inflammatory cytokines (19, 20), B cell maturation (24–26) and ACPA production (24). Ectopic lymphoid structures are found at a higher frequency in RA synovial tissue with higher histological indices of inflammation. Their presence does not correlate with clinical disease activity (21, 22), but was associated with differential response to TNF blockade (7, 27). Most probably, the organization of T and B cells in RA synovitis into specialized lymph node-like structures results from modifications of the normal synovial environment, induced either by chronic inflammation or by mechanisms more upstream in the pathogenesis of RA. These modifications result in local exposure of neo-antigens and local T cell stimulation, as suggested by our data showing that synovial nodular aggregates do not share expanded TCRβ chains between joints from the same patient.

Limitations of the study are intrinsically related to the use of whole synovial tissue in the transcriptomic studies, resulting in signal variations due to heterogeneous cellular compositions and preferential detection of common denominator transcriptomic signals, detected because shared by several cell types. Due to the limited amount of available synovial material, presence of germinal center-like structures could not be confirmed by immunohistochemistry. Single cell approaches will be of interest in further exploring differences in mechanisms of activation of clonally expanded T cells in RA synovitis (4, 28, 29), and whether they correlate with specific patterns of disease severity or response to therapy, thereby resulting in improved patient stratification.

## Data Availability Statement

The datasets presented in this study can be found in online repositories. The names of the repository/repositories and accession number(s) can be found below: https://www.ncbi.nlm.nih.gov/geo/, GSE153015.

## Ethics Statement

The studies involving human participants were reviewed and approved by the ethics committee of the Université catholique de Louvain. The patients/participants provided their written informed consent to participate in this study.

## Author Contributions

BL and PD designed the study. CT, LB, AT, CG, and PD collected the biological samples and clinical data. CT, LV, MC, and JC took part in the experimental procedures. CT, LV, JA, TS, CG, PC, JC, PD, and BL analyzed and interpreted the data. All authors wrote and revised the manuscript. All authors contributed to the article and approved the submitted version.

## Funding

This work was funded by grants from Cap48 (charity supported by the Radio Télévision belge francophone) and WELBIO. Clément Triaille is funded by the Fonds National de la Recherche Scientifique (FNRS— Communauté française de Belgique) and the Fondation Saint-Luc (Cliniques Universitaires Saint-Luc, Brussels). Bernard R. Lauwerys is funded in part by the Fonds National de la Recherche Scientifique.

## Conflict of Interest

The authors declare that the research was conducted in the absence of any commercial or financial relationships that could be construed as a potential conflict of interest.

## Supplementary Material

Supplementary Figure 1 Reproducibility of *TCRB* sequencing experiments. All *TCRB* sequencing experiments were run in duplicates. Typical examples on the Figure (from patient 4) show the variations in reproducibility in function of clonotype frequencies.

Supplementary Figure 2 Overlap between transcripts significantly overexpressed (using moderated t tests on GeneSpring software) in all RA, small RA or large RA compared to OA joints. Numbers of transcripts in each group are displayed on the graph.

Supplementary Table 1 Gene ontology (GO) biological processes significantly enriched in transcripts overexpressed in RA (n=20) compared to OA (n=4) synovial biopsies.

Supplementary Table 2 Transcripts overexpressed in RA (n=20) compared to OA (n=4) synovial biopsy samples and belonging to the following GO pathways: TCR signaling, cellular response to TNF and T cell co stimulation.

Supplementary Table 3 Correlation between TCR signaling gene score, CD3+ histological score and TCRB/RPP30 expression ratio (qPCR).

Supplementary Table 4 Enriched TCRB sequences in RA synovial biopsies.
