# Supplementary figures and images for: Paired Rheumatoid Arthritis Synovial Biopsies From Small and Large Joints Show Similar Global Transcriptomic Patterns With Enrichment of Private Specificity TCRB and TCR Signaling Pathways
Source: Front Immunol. 2020 Nov 23;11:593083. doi: 10.3389/fimmu.2020.593083 (PMC7719799; doi:10.3389/fimmu.2020.593083)

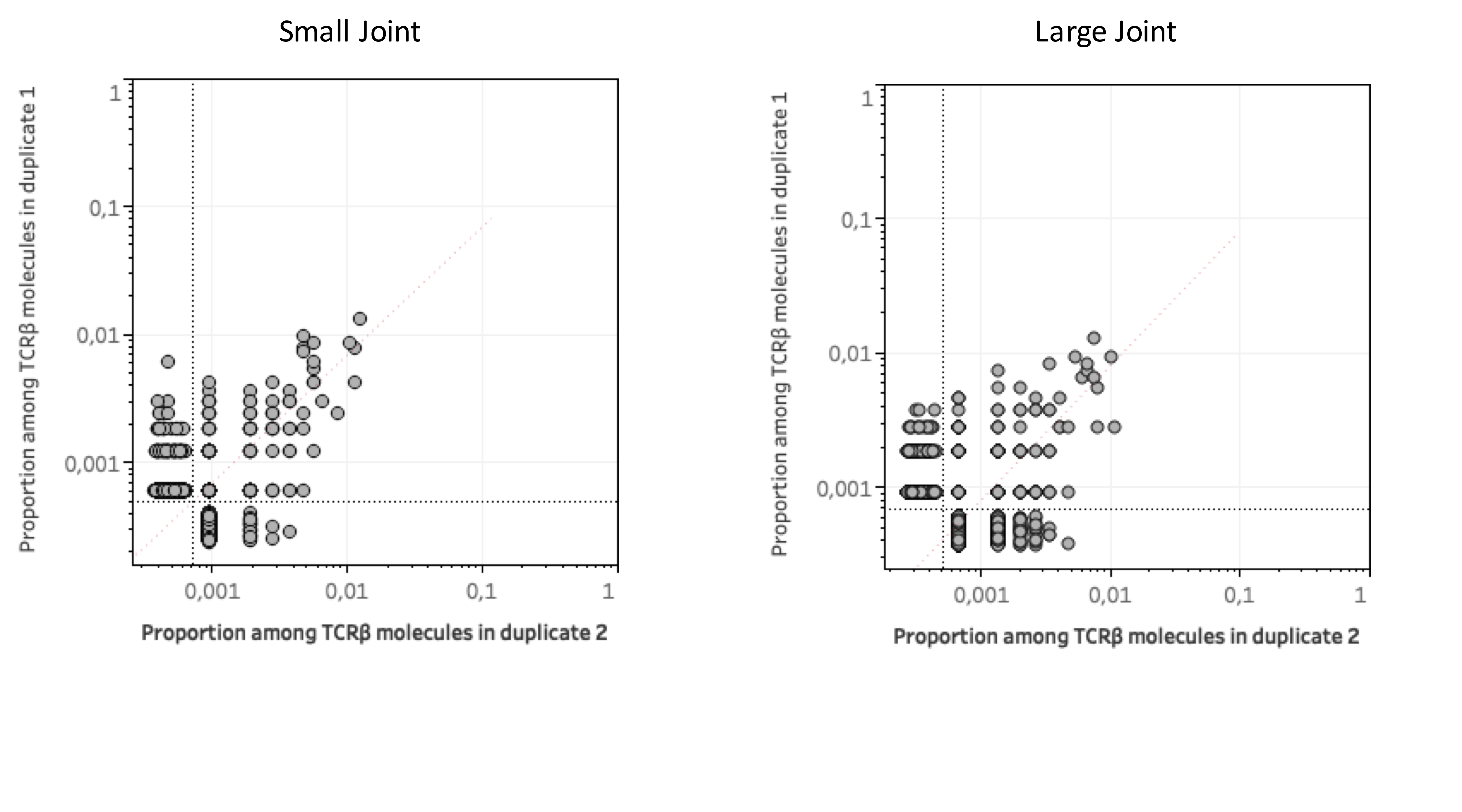

Supplement: Supplementary Figure 1 — Reproducibility of TCRB sequencing experiments. All TCRB sequencing experiments were run in duplicates. Typical examples on the Figure (from patient 4) show the variations in reproducibility in function of clonotype frequencies. [file Image_1.tiff]

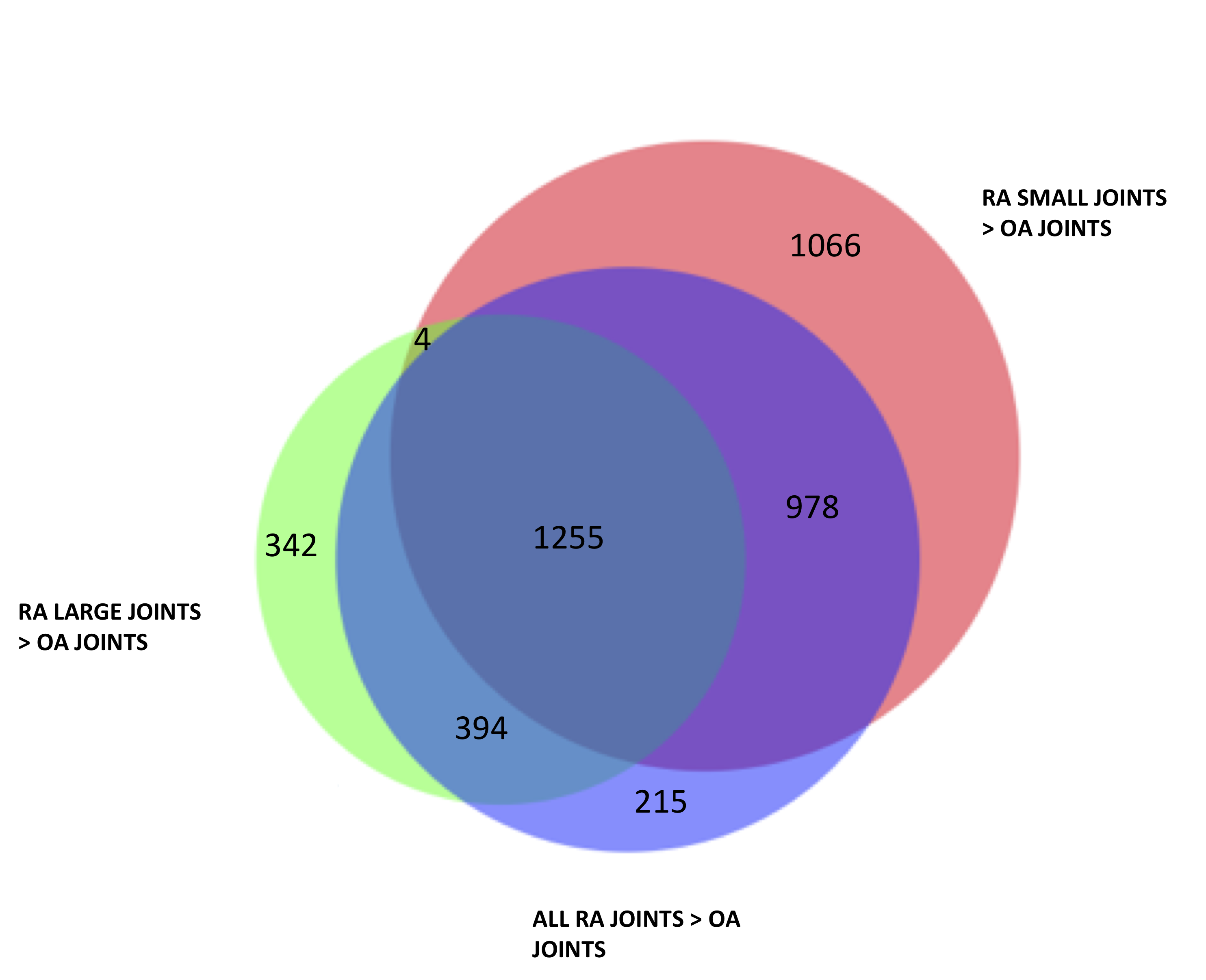

Supplement: Supplementary Figure 2 — Overlap between transcripts significantly overexpressed (using moderated t tests on GeneSpring software) in all RA, small RA or large RA compared to OA joints. Numbers of transcripts in each group are displayed on the graph. [file Image_2.tiff]
