# Supplementary material for: Paired Rheumatoid Arthritis Synovial Biopsies From Small and Large Joints Show Similar Global Transcriptomic Patterns With Enrichment of Private Specificity TCRB and TCR Signaling Pathways
Source: Front Immunol. 2020 Nov 23;11:593083. doi: 10.3389/fimmu.2020.593083 (PMC7719799; doi:10.3389/fimmu.2020.593083)
Supplement: Supplementary Table 1 — Gene ontology (GO) biological processes significantly enriched in transcripts overexpressed in RA (n=20) compared to OA (n=4) synovial biopsies. [file DataSheet_1.pdf]

Supplementary Table 1: Gene ontology (GO) biological processes significantly enriched (corrected p-value < 0.01) in 2,842 transcripts overexpressed in RA (n=20) compared to OA (n=4) synovial biopsies.

GO ACCESSION (BIOLOGICAL PROCESS)	GO Term	p-value	corrected p-value	Count in Selection	% Count in Selection	Count in Total	% Count in Total
GO:0006955	immune response	2,73E-41	1,39E-36	350	17,89366	1651	8,895953
GO:0044428	nuclear part	1,04E-40	3,53E-36	690	35,276073	4229	22,786789
GO:0002376	immune system process	2,65E-38	5,40E-34	452	23,108385	2432	13,104155
GO:0051249	regulation of lymphocyte activation	4,99E-30	5,66E-26	145	7,413088	511	2,753381
GO:0050851	antigen receptor-mediated signaling pathway	1,03E-28	1,05E-24	98	5,010225	282	1,5194784
GO:0050776	regulation of immune response	2,11E-28	1,96E-24	247	12,627811	1172	6,3149953
GO:0050778	positive regulation of immune response	3,68E-28	3,13E-24	181	9,253579	750	4,041166
GO:0002684	positive regulation of immune system process	9,27E-28	6,75E-24	226	11,554193	1043	5,619915
GO:0045087|GO:0002226	innate immune response	1,01E-27	6,89E-24	246	12,576687	1177	6,3419366
GO:0051251	positive regulation of lymphocyte activation	5,30E-27	3,18E-23	116	5,9304705	382	2,0583005
GO:0002694	regulation of leukocyte activation	2,38E-26	1,35E-22	149	7,617587	574	3,092839
GO:0002757	immune response-activating signal transduction	3,52E-26	1,89E-22	137	7,00409	512	2,7587693
GO:0002682	regulation of immune system process	1,41E-25	6,87E-22	318	16,25767	1726	9,30007
GO:0043170|GO:0043283|GO:0044259	macromolecule metabolic process	2,17E-25	1,01E-21	1042	53,271984	7841	42,249043
GO:0002252	immune effector process	2,89E-25	1,28E-21	150	7,6687117	593	3,1952152
GO:0002696	positive regulation of leukocyte activation	3,53E-25	1,44E-21	118	6,0327196	410	2,2091708
GO:0050867	positive regulation of cell activation	3,50E-25	1,44E-21	120	6,134969	421	2,2684412
GO:0050865	regulation of cell activation	6,03E-25	2,37E-21	152	7,7709613	609	3,281427
GO:0002429	immune response-activating cell surface receptor signaling path	6,27E-25	2,37E-21	114	5,828221	396	2,1337357
GO:0050871	positive regulation of B cell activation	7,37E-25	2,69E-21	67	3,4253578	160	0,8621154
GO:0002253	activation of immune response	7,91E-24	2,78E-20	141	7,208589	563	3,0335686
GO:0050864	regulation of B cell activation	3,32E-23	1,13E-19	74	3,783231	201	1,0830325
GO:0002764	immune response-regulating signaling pathway	5,14E-23	1,69E-19	162	8,282208	703	3,7879195
GO:0006952|GO:0002217|GO:0042829	defense response	8,65E-23	2,76E-19	326	16,666666	1851	9,973598
GO:0019221	cytokine-mediated signaling pathway	2,13E-22	6,39E-19	136	6,9529653	551	2,96891
GO:0044446	intracellular organelle part	4,26E-22	1,24E-18	1080	55,214725	8343	44,95393
GO:0050853	B cell receptor signaling pathway	1,09E-21	3,01E-18	55	2,811861	126	0,6789159
GO:0002768	immune response-regulating cell surface receptor signaling path	5,05E-21	1,36E-17	141	7,208589	600	3,2329328
GO:0000278|GO:0007067	mitotic cell cycle	1,23E-20	3,13E-17	179	9,151329	851	4,5853763
GO:0006910	phagocytosis, recognition	1,04E-19	2,46E-16	47	2,402863	103	0,5549868
GO:0002250	adaptive immune response	1,58E-19	3,66E-16	100	5,1124744	368	1,9828655
GO:0006807	nitrogen compound metabolic process	2,71E-19	6,14E-16	1096	56,03272	8639	46,548843
GO:0006950	response to stress	2,77E-19	6,15E-16	581	29,703476	4009	21,60138
GO:0044422	organelle part	5,69E-19	1,24E-15	1085	55,47035	8542	46,026188
GO:0009987|GO:0008151|GO:0044763|GO:00[illegible]	cellular process	1,49E-18	3,04E-15	1648	84,25358	14218	76,60973
GO:0046649	lymphocyte activation	3,68E-18	7,07E-15	104	5,316973	407	2,193006
GO:0044260|GO:0034960	cellular macromolecule metabolic process	4,73E-18	8,93E-15	884	45,194275	6727	36,246567
GO:0044424	intracellular part	1,02E-17	1,82E-14	1601	81,850716	13752	74,09882
GO:0048584	positive regulation of response to stimulus	1,41E-17	2,43E-14	351	17,944786	2189	11,794817
GO:0060337	type I interferon signaling pathway	1,51E-17	2,52E-14	36	1,8404908	70	0,3771755
GO:0071357	cellular response to type I interferon	1,51E-17	2,52E-14	36	1,8404908	70	0,3771755

GO:0044238	primary metabolic process	1,58E-17	2,61E-14	1136	58,07771	9101	49,038204
GO:0071345	cellular response to cytokine stimulus	1,88E-17	3,04E-14	149	7,617587	707	3,8094726
GO:0038095	Fc-epsilon receptor signaling pathway	2,77E-17	4,41E-14	101	5,163599	406	2,1876178
GO:0045321	leukocyte activation	3,25E-17	5,09E-14	115	5,8793454	486	2,6186755
GO:0007049	cell cycle	3,55E-17	5,49E-14	245	12,525562	1390	7,489628
GO:0034097	response to cytokine	3,74E-17	5,70E-14	165	8,435583	820	4,4183416
GO:0034340	response to type I interferon	8,78E-17	1,28E-13	36	1,8404908	73	0,3933402
GO:0038093	Fc receptor signaling pathway	9,82E-17	1,41E-13	110	5,623722	468	2,5216875
GO:0002449	lymphocyte mediated immunity	1,49E-16	2,11E-13	68	3,4764826	222	1,1961851
GO:0006911	phagocytosis, engulfment	1,69E-16	2,35E-13	45	2,3006134	111	0,5980926
GO:1903047	mitotic cell cycle process	2,10E-16	2,85E-13	132	6,7484665	613	3,3029797
GO:0019538|GO:0006411|GO:0044268	protein metabolic process	3,88E-16	5,14E-13	634	32,41309	4598	24,775042
GO:0043207	response to external biotic stimulus	4,34E-16	5,61E-13	188	9,611452	995	5,3612804
GO:0051707|GO:0009613|GO:0042828	response to other organism	4,34E-16	5,61E-13	188	9,611452	995	5,3612804
GO:0048583	regulation of response to stimulus	6,43E-16	8,20E-13	567	28,98773	4048	21,81152
GO:0002460	adaptive immune response based on somatic recombination of i	6,78E-16	8,54E-13	68	3,4764826	228	1,2285144
GO:0051276|GO:0007001|GO:0051277	chromosome organization	6,91E-16	8,59E-13	192	9,815951	1028	5,5390916
GO:0044267	cellular protein metabolic process	1,06E-15	1,30E-12	551	28,169735	3904	21,035616
GO:0009607	response to biotic stimulus	1,16E-15	1,41E-12	191	9,764826	1026	5,528315
GO:0071704	organic substance metabolic process	1,20E-15	1,44E-12	1157	59,15133	9415	50,730103
GO:0008152|GO:0044236|GO:0044710	metabolic process	1,24E-15	1,47E-12	1274	65,13293	10539	56,786465
GO:0007166	cell surface receptor signaling pathway	1,60E-15	1,88E-12	392	20,0409	2587	13,939328
GO:0043412	macromolecule modification	1,68E-15	1,94E-12	473	24,182005	3263	17,581766
GO:0044403|GO:0043298|GO:0044404|GO:00	symbiont process	2,37E-15	2,72E-12	164	8,384459	849	4,5745997
GO:0002443|GO:0019723|GO:0042087	leukocyte mediated immunity	2,45E-15	2,78E-12	75	3,8343558	272	1,4655962
GO:0044419	interspecies interaction between organisms	3,68E-15	3,99E-12	166	8,486708	867	4,671588
GO:0006464	cellular protein modification process	3,64E-15	3,99E-12	449	22,95501	3077	16,579557
GO:0036211	protein modification process	3,64E-15	3,99E-12	449	22,95501	3077	16,579557
GO:0099024	plasma membrane invagination	3,75E-15	4,02E-12	45	2,3006134	119	0,6411983
GO:0051704|GO:0051706	multi-organism process	4,05E-15	4,31E-12	392	20,0409	2602	14,020152
GO:0006958	complement activation, classical pathway	4,33E-15	4,56E-12	46	2,3517382	124	0,6681395
GO:0098542	defense response to other organism	4,74E-15	4,94E-12	126	6,4417176	590	3,1790507
GO:0038094	Fc-gamma receptor signaling pathway	4,97E-15	5,13E-12	56	2,8629856	173	0,9321623
GO:0002433	immune response-regulating cell surface receptor signaling path	6,72E-15	6,79E-12	55	2,811861	169	0,9106094
GO:0038096	Fc-gamma receptor signaling pathway involved in phagocytosis	6,72E-15	6,79E-12	55	2,811861	169	0,9106094
GO:0002431	Fc receptor mediated stimulatory signaling pathway	1,18E-14	1,18E-11	55	2,811861	171	0,9213858
GO:0006956	complement activation	2,00E-14	1,98E-11	48	2,4539878	138	0,7435746
GO:0044770	cell cycle phase transition	2,91E-14	2,86E-11	80	4,0899796	319	1,7188426
GO:0010324|GO:1902534	membrane invagination	3,09E-14	3,00E-11	45	2,3006134	125	0,6735277
GO:0050852	T cell receptor signaling pathway	3,20E-14	3,08E-11	56	2,8629856	185	0,9968209
GO:0042110	T cell activation	3,78E-14	3,61E-11	72	3,6809816	268	1,4440433
GO:0019724	B cell mediated immunity	6,06E-14	5,72E-11	55	2,811861	177	0,9537152
GO:0045088	regulation of innate immune response	6,75E-14	6,32E-11	97	4,9591002	428	2,3061588
GO:0050863	regulation of T cell activation	7,26E-14	6,73E-11	84	4,2944784	342	1,8427716
GO:0016064	immunoglobulin mediated immune response	8,32E-14	7,65E-11	54	2,7607362	173	0,9321623

GO:0006259|GO:0055132	DNA metabolic process	1,14E-13	1,04E-10	158	8,077709	836	4,504553
GO:0044772	mitotic cell cycle phase transition	1,24E-13	1,12E-10	78	3,98773	315	1,6972897
GO:0016032|GO:0022415	viral process	1,81E-13	1,62E-10	146	7,464213	763	4,1112127
GO:0050794|GO:0051244	regulation of cellular process	2,04E-13	1,81E-10	1239	63,34356	10339	55,70882
GO:0006909	phagocytosis	2,11E-13	1,85E-10	70	3,578732	265	1,4278786
GO:0002455	humoral immune response mediated by circulating immunoglob	2,24E-13	1,95E-10	46	2,3517382	136	0,7327981
GO:0006996|GO:1902589	organelle organization	2,40E-13	2,08E-10	461	23,568506	3238	17,447062
GO:0090304	nucleic acid metabolic process	5,37E-13	4,52E-10	562	28,732107	4117	22,183308
GO:0044427	chromosomal part	6,80E-13	5,68E-10	153	7,822086	818	4,407565
GO:0048518|GO:0043119	positive regulation of biological process	8,22E-13	6,82E-10	708	36,19632	5428	29,247265
GO:0070647	protein modification by small protein conjugation or removal	1,86E-12	1,51E-09	157	8,026585	864	4,655423
GO:0022402	cell cycle process	1,86E-12	1,51E-09	175	8,94683	995	5,3612804
GO:0048522|GO:0051242	positive regulation of cellular process	2,36E-12	1,87E-09	647	33,07771	4912	26,466944
GO:1903037	regulation of leukocyte cell-cell adhesion	2,89E-12	2,27E-09	77	3,9366052	321	1,729619
GO:0006325|GO:0016568	chromatin organization	3,23E-12	2,52E-09	137	7,00409	720	3,8795195
GO:0044265|GO:0034962	cellular macromolecule catabolic process	3,70E-12	2,86E-09	156	7,97546	864	4,655423
GO:0044237	cellular metabolic process	4,13E-12	3,17E-09	1108	56,646217	9153	49,31839
GO:0001775	cell activation	5,89E-12	4,45E-09	140	7,157464	747	4,0250015
GO:0044451	nucleoplasm part	6,45E-12	4,84E-09	141	7,208589	755	4,068107
GO:0009617|GO:0009618|GO:0009680	response to bacterium	6,81E-12	5,08E-09	127	6,4928427	657	3,5400615
GO:0008037	cell recognition	1,85E-11	1,34E-08	59	3,0163598	224	1,2069616
GO:0051716	cellular response to stimulus	1,85E-11	1,34E-08	821	41,973415	6520	35,131203
GO:0065007	biological regulation	2,08E-11	1,48E-08	1356	69,32516	11616	62,58958
GO:0006959	humoral immune response	2,56E-11	1,81E-08	65	3,3231084	261	1,4063258
GO:0072376	protein activation cascade	2,61E-11	1,83E-08	48	2,4539878	164	0,8836683
GO:0007165|GO:0023033	signal transduction	3,92E-11	2,67E-08	655	33,486706	5044	27,178188
GO:0060333	interferon-gamma-mediated signaling pathway	4,00E-11	2,71E-08	32	1,6359918	85	0,4579988
GO:0006974|GO:0034984	cellular response to DNA damage stimulus	4,56E-11	3,04E-08	144	7,3619633	804	4,33213
GO:0060255	regulation of macromolecule metabolic process	4,66E-11	3,07E-08	762	38,957054	6009	32,377823
GO:0050789|GO:0050791	regulation of biological process	4,67E-11	3,07E-08	1286	65,74642	10951	59,006413
GO:1901564	organonitrogen compound metabolic process	5,83E-11	3,81E-08	714	36,503067	5591	30,125546
GO:0010467	gene expression	6,70E-11	4,35E-08	537	27,453987	4019	21,655262
GO:0031343	positive regulation of cell killing	7,05E-11	4,55E-08	25	1,2781186	56	0,3017404
GO:0071840|GO:0071841	cellular component organization or biogenesis	7,28E-11	4,67E-08	716	36,605316	5615	30,254864
GO:1903039	positive regulation of leukocyte cell-cell adhesion	7,42E-11	4,73E-08	63	3,2208588	255	1,3739965
GO:0031323	regulation of cellular metabolic process	7,76E-11	4,92E-08	775	39,621677	6141	33,089066
GO:0006139|GO:0055134	nucleobase-containing compound metabolic process	1,08E-10	6,72E-08	606	30,981596	4627	24,931301
GO:0019222|GO:0044246	regulation of metabolic process	1,15E-10	7,08E-08	818	41,82004	6544	35,26052
GO:0051607	defense response to virus	1,15E-10	7,08E-08	49	2,5051124	176	0,948327
GO:0045089	positive regulation of innate immune response	1,18E-10	7,19E-08	74	3,783231	332	1,7888895
GO:0032446	protein modification by small protein conjugation	1,23E-10	7,48E-08	137	7,00409	764	4,116601
GO:0009615	response to virus	1,47E-10	8,87E-08	64	3,2719836	265	1,4278786
GO:0080090	regulation of primary metabolic process	1,64E-10	9,71E-08	745	38,087936	5890	31,736624
GO:0042742|GO:0042830	defense response to bacterium	2,04E-10	1,20E-07	73	3,7321064	323	1,7403955
GO:0016043|GO:0044235|GO:0071842	cellular component organization	2,16E-10	1,27E-07	697	35,633945	5474	29,495123

GO:0031341	regulation of cell killing	2,24E-10	1,30E-07	29	1,4826176	76	0,4095048
GO:0002708	positive regulation of lymphocyte mediated immunity	2,26E-10	1,30E-07	32	1,6359918	90	0,4849399
GO:0051171	regulation of nitrogen compound metabolic process	2,30E-10	1,32E-07	730	37,321064	5764	31,057709
GO:0012501|GO:0016244	programmed cell death	2,46E-10	1,40E-07	180	9,202454	1092	5,883938
GO:0050870	positive regulation of T cell activation	2,54E-10	1,44E-07	61	3,1186094	250	1,3470553
GO:0035556|GO:0007242|GO:0007243|GO:002	intracellular signal transduction	3,07E-10	1,72E-07	300	15,337423	2046	11,024301
GO:0030217|GO:0042112|GO:0046652	T cell differentiation	3,13E-10	1,74E-07	42	2,1472392	142	0,7651274
GO:0034641	cellular nitrogen compound metabolic process	3,31E-10	1,83E-07	686	35,071575	5387	29,026348
GO:0002821	positive regulation of adaptive immune response	4,33E-10	2,36E-07	32	1,6359918	92	0,4957164
GO:0009893|GO:0044253	positive regulation of metabolic process	4,42E-10	2,39E-07	445	22,750511	3269	17,614096
GO:0045619	regulation of lymphocyte differentiation	5,02E-10	2,70E-07	46	2,3517382	166	0,8944448
GO:0006915|GO:0006917|GO:0008632	apoptotic process	5,38E-10	2,87E-07	176	8,997955	1072	5,776173
GO:0002824	positive regulation of adaptive immune response based on soma	5,63E-10	2,99E-07	31	1,5848671	88	0,4741635
GO:0001910	regulation of leukocyte mediated cytotoxicity	5,67E-10	3,00E-07	26	1,3292433	65	0,3502344
GO:0008219	cell death	5,70E-10	3,00E-07	184	9,406953	1134	6,110243
GO:0045580	regulation of T cell differentiation	6,07E-10	3,18E-07	40	2,0449898	134	0,7220216
GO:0071346	cellular response to interferon-gamma	7,07E-10	3,68E-07	41	2,0961146	140	0,754351
GO:0031349	positive regulation of defense response	7,91E-10	4,10E-07	88	4,4989777	438	2,360041
GO:0010604	positive regulation of macromolecule metabolic process	8,63E-10	4,45E-07	403	20,603271	2926	15,765936
GO:0044843	cell cycle G1/S phase transition	1,02E-09	5,20E-07	46	2,3517382	175	0,9429388
GO:0000082	G1/S transition of mitotic cell cycle	1,02E-09	5,20E-07	46	2,3517382	175	0,9429388
GO:0030098|GO:0046650	lymphocyte differentiation	1,05E-09	5,31E-07	57	2,9141104	234	1,2608438
GO:0022409	positive regulation of cell-cell adhesion	1,10E-09	5,54E-07	66	3,3742332	290	1,5625842
GO:0046483	heterocycle metabolic process	1,15E-09	5,77E-07	619	31,646217	4814	25,938898
GO:0016569	covalent chromatin modification	1,20E-09	5,98E-07	78	3,98773	368	1,9828655
GO:0051603	proteolysis involved in cellular protein catabolic process	1,21E-09	6,02E-07	102	5,214724	538	2,898863
GO:0000075|GO:0031576|GO:0071779	cell cycle checkpoint	1,25E-09	6,17E-07	55	2,811861	229	1,2339027
GO:0044257	cellular protein catabolic process	1,66E-09	8,13E-07	104	5,316973	555	2,9904628
GO:0033554	cellular response to stress	1,80E-09	8,79E-07	263	13,445807	1777	9,574869
GO:0034341	response to interferon-gamma	1,91E-09	9,29E-07	44	2,2494888	161	0,8675036
GO:0001912	positive regulation of leukocyte mediated cytotoxicity	2,17E-09	1,05E-06	22	1,1247444	51	0,2747993
GO:0016570	histone modification	2,69E-09	1,29E-06	76	3,8854806	361	1,9451479
GO:0002706	regulation of lymphocyte mediated immunity	3,12E-09	1,49E-06	38	1,9427403	130	0,7004688
GO:0002705	positive regulation of leukocyte mediated immunity	3,44E-09	1,63E-06	33	1,6871166	104	0,560375
GO:0031325	positive regulation of cellular metabolic process	3,95E-09	1,87E-06	418	21,370144	3089	16,644217
GO:0050829	defense response to Gram-negative bacterium	4,50E-09	2,11E-06	28	1,4314928	80	0,4310577
GO:0050896|GO:0051869	response to stimulus	5,86E-09	2,73E-06	958	48,977505	7961	42,89563
GO:0051173	positive regulation of nitrogen compound metabolic process	6,20E-09	2,88E-06	393	20,092024	2886	15,550406
GO:0030163|GO:0044254	protein catabolic process	6,24E-09	2,88E-06	109	5,572597	604	3,2544856
GO:0002819	regulation of adaptive immune response	7,00E-09	3,21E-06	39	1,993865	139	0,7489628
GO:0022407	regulation of cell-cell adhesion	7,01E-09	3,21E-06	82	4,1922293	409	2,2037826
GO:0009057|GO:0043285|GO:0044266	macromolecule catabolic process	7,11E-09	3,24E-06	162	8,282208	1008	5,4313273
GO:0001916	positive regulation of T cell mediated cytotoxicity	7,61E-09	3,45E-06	16	0,8179959	30	0,1616466
GO:0006725	cellular aromatic compound metabolic process	7,99E-09	3,61E-06	618	31,595093	4860	26,186756
GO:0016567	protein ubiquitination	8,26E-09	3,71E-06	117	5,981595	665	3,5831673

GO:0048519|GO:0043118	negative regulation of biological process	8,69E-09	3,89E-06	624	31,90184	4925	26,53699
GO:0070663	regulation of leukocyte proliferation	9,40E-09	4,19E-06	55	2,811861	235	1,266232
GO:0002711	positive regulation of T cell mediated immunity	9,90E-09	4,39E-06	20	1,0224949	46	0,2478582
GO:1902579	multi-organism localization	1,02E-08	4,50E-06	18	0,9202454	38	0,2047524
GO:0044766	multi-organism transport	1,02E-08	4,50E-06	18	0,9202454	38	0,2047524
GO:0006396|GO:0006394	RNA processing	1,06E-08	4,63E-06	138	7,055215	816	4,3967886
GO:0006397	mRNA processing	1,08E-08	4,70E-06	88	4,4989777	454	2,4462526
GO:0002521	leukocyte differentiation	1,31E-08	5,68E-06	69	3,5276074	327	1,7619483
GO:0032268	regulation of cellular protein metabolic process	1,35E-08	5,83E-06	348	17,79141	2522	13,589094
GO:0001914	regulation of T cell mediated cytotoxicity	1,64E-08	7,01E-06	17	0,86912066	35	0,1885877
GO:0008150|GO:0000004|GO:0007582|GO:004	biological_process	1,76E-08	7,49E-06	1798	91,92229	16372	88,21596
GO:0019219	regulation of nucleobase-containing compound metabolic proce	1,83E-08	7,79E-06	535	27,351738	4147	22,344954
GO:0010608	posttranscriptional regulation of gene expression	2,05E-08	8,68E-06	88	4,4989777	467	2,5162995
GO:0050670	regulation of lymphocyte proliferation	2,22E-08	9,36E-06	53	2,7096114	228	1,2285144
GO:0018205	peptidyl-lysine modification	2,25E-08	9,46E-06	67	3,4253578	318	1,7134544
GO:1902533|GO:0010740	positive regulation of intracellular signal transduction	2,27E-08	9,50E-06	170	8,691207	1079	5,813891
GO:0051246	regulation of protein metabolic process	2,57E-08	1,07E-05	365	18,660532	2681	14,445822
GO:0032944	regulation of mononuclear cell proliferation	2,60E-08	1,08E-05	53	2,7096114	229	1,2339027
GO:0007154	cell communication	2,67E-08	1,10E-05	687	35,1227	5517	29,726818
GO:0001895	retina homeostasis	2,76E-08	1,13E-05	35	1,789366	123	0,6627512
GO:0002218|GO:0002219	activation of innate immune response	2,76E-08	1,13E-05	57	2,9141104	261	1,4063258
GO:0043632	modification-dependent macromolecule catabolic process	2,79E-08	1,13E-05	89	4,550102	477	2,5701816
GO:0046640	regulation of alpha-beta T cell proliferation	2,81E-08	1,13E-05	18	0,9202454	40	0,2155289
GO:0016071	mRNA metabolic process	2,92E-08	1,17E-05	119	6,0838447	687	3,701708
GO:0010468	regulation of gene expression	2,93E-08	1,17E-05	575	29,396729	4515	24,32782
GO:0002822	regulation of adaptive immune response based on somatic reco	3,06E-08	1,22E-05	36	1,8404908	129	0,6950806
GO:0031570	DNA integrity checkpoint	3,39E-08	1,35E-05	40	2,0449898	158	0,851339
GO:0023052|GO:0023046|GO:0044700	signaling	3,49E-08	1,38E-05	676	34,560326	5426	29,236488
GO:0002483	antigen processing and presentation of endogenous peptide ant	3,54E-08	1,39E-05	10	0,51124746	13	0,0700469
GO:0033209	tumor necrosis factor-mediated signaling pathway	3,97E-08	1,55E-05	39	1,993865	153	0,8243979
GO:0007264	small GTPase mediated signal transduction	4,26E-08	1,66E-05	140	7,157464	857	4,617706
GO:0019941	modification-dependent protein catabolic process	4,69E-08	1,82E-05	87	4,4478526	468	2,5216875
GO:0000077	DNA damage checkpoint	5,64E-08	2,18E-05	38	1,9427403	149	0,802845
GO:1902531|GO:0010627	regulation of intracellular signal transduction	6,53E-08	2,51E-05	251	12,832311	1747	9,413222
GO:0002479	antigen processing and presentation of exogenous peptide antig	6,73E-08	2,58E-05	25	1,2781186	79	0,4256695
GO:0046634	regulation of alpha-beta T cell activation	8,35E-08	3,18E-05	28	1,4314928	90	0,4849399
GO:0050671	positive regulation of lymphocyte proliferation	8,32E-08	3,18E-05	39	1,993865	151	0,8136214
GO:0042113	B cell activation	8,67E-08	3,28E-05	41	2,0961146	163	0,8782801
GO:0006511|GO:0042787|GO:0043432	ubiquitin-dependent protein catabolic process	8,68E-08	3,28E-05	85	4,3456035	460	2,478582
GO:0008380|GO:0006395	RNA splicing	9,56E-08	3,60E-05	76	3,8854806	391	2,1067946
GO:0032946	positive regulation of mononuclear cell proliferation	1,01E-07	3,76E-05	39	1,993865	152	0,8190097
GO:0002703	regulation of leukocyte mediated immunity	1,04E-07	3,89E-05	42	2,1472392	170	0,9159976
GO:0045621	positive regulation of lymphocyte differentiation	1,08E-07	3,99E-05	31	1,5848671	107	0,5765397
GO:0019731|GO:0006961|GO:0019733	antibacterial humoral response	1,21E-07	4,48E-05	25	1,2781186	76	0,4095048
GO:0009967|GO:0035468	positive regulation of signal transduction	1,34E-07	4,95E-05	229	11,707566	1581	8,518778

GO:0016070	RNA metabolic process	1,52E-07	5,54E-05	464	23,721882	3574	19,257504
GO:0051252	regulation of RNA metabolic process	1,55E-07	5,65E-05	494	25,255623	3847	20,728487
GO:0051726|GO:0000074	regulation of cell cycle	1,56E-07	5,66E-05	164	8,384459	1062	5,722291
GO:0002758	innate immune response-activating signal transduction	1,56E-07	5,66E-05	54	2,7607362	254	1,3686082
GO:0006260|GO:0055133	DNA replication	1,74E-07	6,27E-05	48	2,4539878	210	1,1315265
GO:0070665	positive regulation of leukocyte proliferation	1,76E-07	6,32E-05	39	1,993865	155	0,8351743
GO:0042590	antigen processing and presentation of exogenous peptide antig	2,06E-07	7,35E-05	25	1,2781186	83	0,4472224
GO:0080134	regulation of response to stress	2,17E-07	7,71E-05	213	10,88957	1468	7,909909
GO:2000113	negative regulation of cellular macromolecule biosynthetic proc	2,26E-07	7,96E-05	196	10,02045	1315	7,085511
GO:0009894	regulation of catabolic process	2,32E-07	8,16E-05	144	7,3619633	912	4,9140577
GO:0019885|GO:0048004	antigen processing and presentation of endogenous peptide ant	2,60E-07	9,12E-05	9	0,4601227	12	0,0646587
GO:0031347	regulation of defense response	2,63E-07	9,17E-05	136	6,9529653	852	4,5907645
GO:0018193	peptidyl-amino acid modification	2,94E-07	1,02E-04	158	8,077709	1025	5,522927
GO:1901360	organic cyclic compound metabolic process	3,02E-07	1,04E-04	629	32,157463	5072	27,32906
GO:0019883	antigen processing and presentation of endogenous antigen	3,05E-07	1,05E-04	10	0,51124746	15	0,0808233
GO:0032816	positive regulation of natural killer cell activation	3,46E-07	1,18E-04	15	0,76687115	33	0,1778113
GO:0019730|GO:0006960|GO:0019735	antimicrobial humoral response	3,70E-07	1,26E-04	25	1,2781186	80	0,4310577
GO:0002200	somatic diversification of immune receptors	3,75E-07	1,27E-04	18	0,9202454	46	0,2478582
GO:0010556	regulation of macromolecule biosynthetic process	3,86E-07	1,30E-04	518	26,482618	4078	21,973167
GO:0044454	nuclear chromosome part	4,17E-07	1,39E-04	89	4,550102	498	2,683334
GO:0031329	regulation of cellular catabolic process	5,18E-07	1,72E-04	129	6,595092	808	4,353683
GO:0050792	regulation of viral process	5,47E-07	1,81E-04	46	2,3517382	205	1,1045854
GO:0046641	positive regulation of alpha-beta T cell proliferation	5,58E-07	1,84E-04	15	0,76687115	34	0,1831995
GO:2000112	regulation of cellular macromolecule biosynthetic process	6,10E-07	2,01E-04	505	25,817995	3977	21,428957
GO:0031295	T cell costimulation	6,66E-07	2,19E-04	29	1,4826176	104	0,560375
GO:0045582	positive regulation of T cell differentiation	6,69E-07	2,19E-04	27	1,3803681	93	0,5011046
GO:0010605	negative regulation of macromolecule metabolic process	6,96E-07	2,27E-04	335	17,12679	2498	13,459777
GO:0016458	gene silencing	7,48E-07	2,43E-04	48	2,4539878	220	1,1854087
GO:0006897|GO:0016193|GO:0016196|GO:009	endocytosis	7,76E-07	2,50E-04	105	5,3680983	631	3,3999677
GO:0010558	negative regulation of macromolecule biosynthetic process	7,83E-07	2,52E-04	202	10,327198	1387	7,473463
GO:0031294	lymphocyte costimulation	8,29E-07	2,66E-04	29	1,4826176	105	0,5657632
GO:0045862	positive regulation of proteolysis	9,39E-07	3,00E-04	64	3,2719836	329	1,7727249
GO:0002520	immune system development	9,94E-07	3,16E-04	111	5,6748466	672	3,6208847
GO:0043900	regulation of multi-organism process	9,97E-07	3,16E-04	86	4,396728	486	2,6186755
GO:0009892|GO:0044252	negative regulation of metabolic process	1,07E-06	3,39E-04	361	18,456032	2733	14,726009
GO:0044783	G1 DNA damage checkpoint	1,12E-06	3,52E-04	23	1,1758691	79	0,4256695
GO:0033151	V(D)J recombination	1,19E-06	3,73E-04	11	0,5623722	20	0,1077644
GO:0061013	regulation of mRNA catabolic process	1,20E-06	3,73E-04	37	1,8916155	160	0,8621154
GO:0045069	regulation of viral genome replication	1,19E-06	3,73E-04	23	1,1758691	74	0,3987284
GO:0010647	positive regulation of cell communication	1,25E-06	3,87E-04	240	12,269938	1718	9,256965
GO:0002709	regulation of T cell mediated immunity	1,28E-06	3,96E-04	21	1,0736196	64	0,3448462
GO:0043122	regulation of I-kappaB kinase/NF-kappaB signaling	1,29E-06	3,98E-04	46	2,3517382	211	1,1369147
GO:0002474	antigen processing and presentation of peptide antigen via MHC	1,35E-06	4,14E-04	29	1,4826176	113	0,608869
GO:0043903	regulation of symbiosis, encompassing mutualism through paras	1,37E-06	4,21E-04	49	2,5051124	231	1,2446791
GO:0034612	response to tumor necrosis factor	1,39E-06	4,24E-04	54	2,7607362	271	1,4602079

GO:0002699	positive regulation of immune effector process	1,41E-06	4,28E-04	42	2,1472392	186	1,0022092
GO:0061024|GO:0016044|GO:0044802	membrane organization	1,49E-06	4,52E-04	129	6,595092	816	4,3967886
GO:0045071	negative regulation of viral genome replication	1,59E-06	4,79E-04	18	0,9202454	50	0,2694111
GO:0023056	positive regulation of signaling	1,63E-06	4,92E-04	240	12,269938	1724	9,289293
GO:0046635	positive regulation of alpha-beta T cell activation	1,64E-06	4,93E-04	22	1,1247444	70	0,3771755
GO:0048523|GO:0051243	negative regulation of cellular process	1,81E-06	5,41E-04	539	27,556236	4333	23,347162
GO:0006310	DNA recombination	1,85E-06	5,50E-04	57	2,9141104	287	1,5464195
GO:0010950	positive regulation of endopeptidase activity	1,94E-06	5,72E-04	38	1,9427403	163	0,8782801
GO:0031324	negative regulation of cellular metabolic process	1,94E-06	5,72E-04	314	16,05317	2345	12,635379
GO:0006281	DNA repair	2,17E-06	6,33E-04	91	4,652352	532	2,8665338
GO:0060249	anatomical structure homeostasis	2,20E-06	6,40E-04	65	3,3231084	344	1,8535482
GO:1902105	regulation of leukocyte differentiation	2,23E-06	6,45E-04	55	2,811861	275	1,4817609
GO:0002825	regulation of T-helper 1 type immune response	2,37E-06	6,85E-04	14	0,7157464	33	0,1778113
GO:0031326	regulation of cellular biosynthetic process	2,46E-06	7,10E-04	532	27,198364	4263	22,969988
GO:0043067|GO:0043070	regulation of programmed cell death	2,72E-06	7,78E-04	213	10,88957	1511	8,1416025
GO:0043123	positive regulation of I-kappaB kinase/NF-kappaB signaling	2,96E-06	8,44E-04	39	1,993865	172	0,9267741
GO:0032479	regulation of type I interferon production	3,02E-06	8,58E-04	32	1,6359918	129	0,6950806
GO:0031327	negative regulation of cellular biosynthetic process	3,17E-06	8,98E-04	209	10,685072	1473	7,93685
GO:0042129	regulation of T cell proliferation	3,27E-06	9,21E-04	40	2,0449898	179	0,9644916
GO:0031571	mitotic G1 DNA damage checkpoint	3,29E-06	9,25E-04	22	1,1247444	78	0,4202813
GO:0006261|GO:0006262|GO:0006263	DNA-dependent DNA replication	3,33E-06	9,35E-04	28	1,4314928	106	0,5711514
GO:0042981	regulation of apoptotic process	3,35E-06	9,37E-04	211	10,787321	1499	8,076943
GO:0009889	regulation of biosynthetic process	3,51E-06	9,75E-04	536	27,402863	4311	23,228622
GO:0050790|GO:0048552	regulation of catalytic activity	3,69E-06	0,001022041	318	16,25767	2405	12,958673
GO:0044819	mitotic G1/S transition checkpoint	4,18E-06	0,00115306	22	1,1247444	79	0,4256695
GO:0002480	antigen processing and presentation of exogenous peptide antig	4,28E-06	0,00117623	7	0,3578732	9	0,048494
GO:0098657	import into cell	4,51E-06	0,001229667	108	5,5214725	678	3,653214
GO:0002639	positive regulation of immunoglobulin production	4,53E-06	0,001232454	15	0,76687115	39	0,2101406
GO:0009890	negative regulation of biosynthetic process	4,96E-06	0,001338934	210	10,7361965	1491	8,033838
GO:1903708	positive regulation of hemopoiesis	5,07E-06	0,001365908	42	2,1472392	195	1,0507032
GO:0002697	regulation of immune effector process	5,22E-06	0,001401858	76	3,8854806	432	2,3277116
GO:0032655	regulation of interleukin-12 production	5,33E-06	0,001427711	20	1,0224949	64	0,3448462
GO:0010498	proteasomal protein catabolic process	5,55E-06	0,001483068	61	3,1186094	332	1,7888895
GO:1901991	negative regulation of mitotic cell cycle phase transition	5,65E-06	0,001504623	37	1,8916155	170	0,9159976
GO:0031047	gene silencing by RNA	5,76E-06	0,001531464	34	1,7382413	145	0,7812921
GO:0051172	negative regulation of nitrogen compound metabolic process	5,94E-06	0,001573576	293	14,97955	2195	11,827146
GO:0010952	positive regulation of peptidase activity	6,16E-06	0,001624927	39	1,993865	177	0,9537152
GO:1901988	negative regulation of cell cycle phase transition	6,16E-06	0,001624927	38	1,9427403	177	0,9537152
GO:1903706	regulation of hemopoiesis	6,36E-06	0,001671569	67	3,4253578	369	1,9882537
GO:0030330|GO:0006976	DNA damage response, signal transduction by p53 class mediatc	6,38E-06	0,001672756	24	1,2269939	92	0,4957164
GO:0043281|GO:0043026	regulation of cysteine-type endopeptidase activity involved in ap	6,52E-06	0,001703065	47	2,402863	230	1,239291
GO:0016444	somatic cell DNA recombination	6,54E-06	0,001703065	15	0,76687115	40	0,2155289
GO:0002562	somatic diversification of immune receptors via germline recom	6,54E-06	0,001703065	15	0,76687115	40	0,2155289
GO:2000116	regulation of cysteine-type endopeptidase activity	6,93E-06	0,001795446	49	2,5051124	244	1,314726
GO:0002827	positive regulation of T-helper 1 type immune response	7,01E-06	0,001810012	12	0,61349696	27	0,145482

GO:0097296	activation of cysteine-type endopeptidase activity involved in ap	7,09E-06	0,001827604	11	0,5623722	23	0,1239291
GO:1902107	positive regulation of leukocyte differentiation	7,26E-06	0,001865701	36	1,8404908	159	0,8567272
GO:0042098|GO:0042111	T cell proliferation	7,34E-06	0,001881204	19	0,97137016	60	0,3232933
GO:0051301	cell division	7,55E-06	0,001930558	85	4,3456035	503	2,7102754
GO:0001817	regulation of cytokine production	7,68E-06	0,001959626	102	5,214724	632	3,405356
GO:0051247	positive regulation of protein metabolic process	8,15E-06	0,002073773	226	11,554193	1644	8,858236
GO:1902583	multi-organism intracellular transport	8,72E-06	0,00220812	6	0,30674848	7	0,0377176
GO:1902581	multi-organism cellular localization	8,72E-06	0,00220812	6	0,30674848	7	0,0377176
GO:1903311	regulation of mRNA metabolic process	8,93E-06	0,002256072	49	2,5051124	253	1,36322
GO:0010629	negative regulation of gene expression	8,96E-06	0,002256072	234	11,96319	1705	9,186917
GO:0034645|GO:0034961	cellular macromolecule biosynthetic process	8,99E-06	0,002258269	475	24,284254	3799	20,469852
GO:0032814	regulation of natural killer cell activation	9,32E-06	0,002335357	15	0,76687115	41	0,2209171
GO:0072676	lymphocyte migration	1,01E-05	0,002514422	18	0,9202454	56	0,3017404
GO:0090305	nucleic acid phosphodiester bond hydrolysis	1,04E-05	0,002591259	54	2,7607362	282	1,5194784
GO:0000375|GO:0000385|GO:0031202	RNA splicing, via transesterification reactions	1,04E-05	0,002591259	54	2,7607362	282	1,5194784
GO:0010533|GO:0010532|GO:0010534	regulation of activation of Janus kinase activity	1,14E-05	0,002818712	10	0,51124746	20	0,1077644
GO:0006919	activation of cysteine-type endopeptidase activity involved in ap	1,18E-05	0,002912762	25	1,2781186	95	0,5118811
GO:0009966|GO:0035466	regulation of signal transduction	1,20E-05	0,002947686	383	19,580776	3013	16,23471
GO:0002220	innate immune response activating cell surface receptor signalin	1,20E-05	0,002956393	30	1,5337423	131	0,705857
GO:0071356	cellular response to tumor necrosis factor	1,23E-05	0,003023939	48	2,4539878	249	1,341667
GO:0042770	signal transduction in response to DNA damage	1,24E-05	0,003045359	26	1,3292433	107	0,5765397
GO:0043161	proteasome-mediated ubiquitin-dependent protein catabolic pr	1,27E-05	0,003091979	57	2,9141104	312	1,681125
GO:0032480	negative regulation of type I interferon production	1,37E-05	0,003325778	16	0,8179959	47	0,2532464
GO:0006898	receptor-mediated endocytosis	1,41E-05	0,003416022	57	2,9141104	306	1,6487957
GO:0048525	negative regulation of viral process	1,44E-05	0,003472515	25	1,2781186	96	0,5172693
GO:0044444	cytoplasmic part	1,44E-05	0,003483361	962	49,182003	8306	44,754566
GO:0046651	lymphocyte proliferation	1,48E-05	0,003551025	26	1,3292433	102	0,5495986
GO:2000134	negative regulation of G1/S transition of mitotic cell cycle	1,49E-05	0,003588446	26	1,3292433	108	0,5819279
GO:0043487	regulation of RNA stability	1,53E-05	0,003668553	32	1,6359918	145	0,7812921
GO:0000398|GO:0006374|GO:0006375	mRNA splicing, via spliceosome	1,58E-05	0,003770337	53	2,7096114	279	1,5033138
GO:0000377	RNA splicing, via transesterification reactions with bulged adeno	1,58E-05	0,003770337	53	2,7096114	279	1,5033138
GO:0043901	negative regulation of multi-organism process	1,60E-05	0,003803586	35	1,789366	158	0,851339
GO:0070265	necrotic cell death	1,68E-05	0,003992364	14	0,7157464	38	0,2047524
GO:0032819	positive regulation of natural killer cell proliferation	1,75E-05	0,00415315	9	0,4601227	17	0,0915998
GO:0048534	hematopoietic or lymphoid organ development	1,81E-05	0,004279187	101	5,163599	637	3,432297
GO:0045935	positive regulation of nucleobase-containing compound metabo	1,82E-05	0,004287377	226	11,554193	1655	8,917506
GO:0071310	cellular response to organic substance	1,88E-05	0,004419676	293	14,97955	2244	12,091168
GO:0006403	RNA localization	1,93E-05	0,004524163	40	2,0449898	192	1,0345385
GO:0010941	regulation of cell death	1,93E-05	0,004524163	219	11,196319	1606	8,653483
GO:0032270	positive regulation of cellular protein metabolic process	2,00E-05	0,004638767	212	10,838446	1548	8,340966
GO:0002223	stimulatory C-type lectin receptor signaling pathway	2,02E-05	0,004673039	29	1,4826176	128	0,6896923
GO:0007093|GO:0031575|GO:0071780	mitotic cell cycle checkpoint	2,13E-05	0,004906495	36	1,8404908	173	0,9321623
GO:0032943	mononuclear cell proliferation	2,14E-05	0,004908217	26	1,3292433	104	0,560375
GO:0006417|GO:0006445	regulation of translation	2,19E-05	0,005029395	60	3,0674846	332	1,7888895
GO:0044093	positive regulation of molecular function	2,32E-05	0,005316562	250	12,781186	1873	10,092138

GO:0032735	positive regulation of interleukin-12 production	2,49E-05	0,005674138	15	0,76687115	44	0,2370817
GO:0043488	regulation of mRNA stability	2,53E-05	0,005755283	31	1,5848671	142	0,7651274
GO:1903050	regulation of proteolysis involved in cellular protein catabolic pr	2,54E-05	0,005765875	45	2,3006134	228	1,2285144
GO:1902807	negative regulation of cell cycle G1/S phase transition	2,54E-05	0,005765875	26	1,3292433	111	0,5980926
GO:0040029	regulation of gene expression, epigenetic	2,64E-05	0,005963578	48	2,4539878	249	1,341667
GO:2001056	positive regulation of cysteine-type endopeptidase activity	2,77E-05	0,006254121	33	1,6871166	149	0,802845
GO:0046637	regulation of alpha-beta T cell differentiation	2,90E-05	0,006535257	18	0,9202454	60	0,3232933
GO:0045934	negative regulation of nucleobase-containing compound metab	2,95E-05	0,006624788	193	9,867076	1390	7,489628
GO:0006468	protein phosphorylation	3,02E-05	0,006769492	149	7,617587	1036	5,582197
GO:0042269	regulation of natural killer cell mediated cytotoxicity	3,04E-05	0,006805077	13	0,66462165	35	0,1885877
GO:0044773	mitotic DNA damage checkpoint	3,05E-05	0,006815808	25	1,2781186	106	0,5711514
GO:0043280	positive regulation of cysteine-type endopeptidase activity invol	3,09E-05	0,00687593	31	1,5848671	137	0,7381863
GO:0032817	regulation of natural killer cell proliferation	3,18E-05	0,007037686	9	0,4601227	18	0,096988
GO:0072678	T cell migration	3,18E-05	0,007037686	9	0,4601227	18	0,096988
GO:0010536|GO:0010535|GO:0010537	positive regulation of activation of Janus kinase activity	3,18E-05	0,007037686	9	0,4601227	18	0,096988
GO:0044417	translocation of molecules into host	3,24E-05	0,007113154	7	0,3578732	11	0,0592704
GO:0051836	translocation of molecules into other organism involved in symb	3,24E-05	0,007113154	7	0,3578732	11	0,0592704
GO:0022616	DNA strand elongation	3,30E-05	0,00720658	14	0,7157464	40	0,2155289
GO:0030890	positive regulation of B cell proliferation	3,30E-05	0,00720658	14	0,7157464	40	0,2155289
GO:2000514	regulation of CD4-positive, alpha-beta T cell activation	3,30E-05	0,00720658	16	0,8179959	50	0,2694111
GO:0045785	positive regulation of cell adhesion	3,39E-05	0,007371399	74	3,783231	440	2,3708174
GO:0030097	hemopoiesis	3,48E-05	0,007549661	93	4,754601	585	3,1521094
GO:0045786	negative regulation of cell cycle	3,68E-05	0,007970737	85	4,3456035	532	2,8665338
GO:0046631	alpha-beta T cell activation	3,71E-05	0,008028024	18	0,9202454	61	0,3286815
GO:0000209	protein polyubiquitination	3,97E-05	0,008560595	44	2,2494888	232	1,2500674
GO:0009059|GO:0043284	macromolecule biosynthetic process	4,02E-05	0,008654851	476	24,335379	3862	20,80931
GO:0043085|GO:0048554	positive regulation of catalytic activity	4,17E-05	0,008958312	214	10,940696	1583	8,529554
GO:0002715|GO:0045845	regulation of natural killer cell mediated immunity	4,30E-05	0,009201546	13	0,66462165	36	0,193976
GO:0010033	response to organic substance	4,31E-05	0,009201546	369	18,86503	2932	15,798265
GO:0097300	programmed necrotic cell death	4,30E-05	0,009201546	13	0,66462165	36	0,193976
GO:1903506	regulation of nucleic acid-templated transcription	4,41E-05	0,009394966	451	23,05726	3642	19,623901
GO:0000186|GO:0007255	activation of MAPKK activity	4,47E-05	0,009494099	47	2,402863	254	1,3686082
GO:0044839	cell cycle G2/M phase transition	4,52E-05	0,00952957	32	1,6359918	146	0,7866803
GO:0000086	G2/M transition of mitotic cell cycle	4,52E-05	0,00952957	32	1,6359918	146	0,7866803
GO:0051028	mRNA transport	4,52E-05	0,00952957	32	1,6359918	146	0,7866803
GO:0051253	negative regulation of RNA metabolic process	4,49E-05	0,00952957	175	8,94683	1250	6,7352767
GO:2001141	regulation of RNA biosynthetic process	4,54E-05	0,009552071	452	23,108385	3652	19,677784
GO:0051254	positive regulation of RNA metabolic process	4,57E-05	0,009584816	200	10,224949	1459	7,861415
GO:0006357|GO:0006358|GO:0010551	regulation of transcription by RNA polymerase II	4,70E-05	0,009838434	249	12,730062	1875	10,102915
