# Supplementary material for: Paired Rheumatoid Arthritis Synovial Biopsies From Small and Large Joints Show Similar Global Transcriptomic Patterns With Enrichment of Private Specificity TCRB and TCR Signaling Pathways
Source: Front Immunol. 2020 Nov 23;11:593083. doi: 10.3389/fimmu.2020.593083 (PMC7719799; doi:10.3389/fimmu.2020.593083)
Supplement: Supplementary Table 2 — Transcripts overexpressed in RA (n=20) compared to OA (n=4) synovial biopsy samples and belonging to the following GO pathways: TCR signaling, cellular response to TNF and T cell co stimulation. [file DataSheet_2.pdf]

Supplementary Table 2: Transcripts overexpressed in RA (n=20) compared to OA (n=4) synovial biopsy samples and belonging to the following GO pathways: TCR signaling, cellular response to TNF and T cell co stimulation. TCR signaling transcripts are ranked in the same order as they appear in Figure 2B.

TCR-SIGNALING PATHWAY

Probe Set ID	Gene Symbol
200039_s_at	PSMB2
200814_at	PSME1
201316_at	PSMA2
201317_s_at	PSMA2
201400_at	PSMB3
201524_x_at	UBE2N
201762_s_at	PSME2
202243_s_at	PSMB4
202352_s_at	PSMD12
202659_at	PSMB10
202753_at	PSMD6
203396_at	PSMA4
203879_at	PIK3CD
203882_at	IRF9
204279_at	PSMB9
204613_at	PLCG2
204890_s_at	LCK
204891_s_at	LCK
205269_at	LCP2
205270_s_at	LCP2
206804_at	CD3G
206854_s_at	MAP3K7
207238_s_at	PTPRC
207485_x_at	BTN3A1
208309_s_at	MALT1
208805_at	KIAA0391///PSMA6
208877_at	PAK2
209040_s_at	PSMB8
209282_at	PRKD2
209341_s_at	IKBKB
209602_s_at	GATA3
209603_at	GATA3
209604_s_at	GATA3
209670_at	TRAC
209671_x_at	TRAC
209881_s_at	LAT
210031_at	CD247
210915_x_at	TRBC1///TRBV19
210972_x_at	TRAC///TRAJ17///TRAV20///TRDV2
211005_at	LAT
211339_s_at	ITK
211764_s_at	UBE2D1
211795_s_at	FYB
211796_s_at	TRBC1///TRBC2///TRBV19///TRBV3-1///TRBV5-4///TRBV6-5///TRBV7-2
211902_x_at	YME1L1
212587_s_at	PTPRC
212588_at	PTPRC
212646_at	RFTN1
213193_x_at	TRBC1///TRBV19
213539_at	CD3D
213830_at	YME1L1

214032_at	ZAP70
214590_s_at	UBE2D1
215536_at	HLA-DQB2
216191_s_at	TRDV3
217143_s_at	TRDC
217147_s_at	TRAT1
38269_at	PRKD2
225622_at	PAG1
225626_at	PAG1
227266_s_at	FYB
227354_at	PAG1
230917_at	PLCG2
244251_at	LCP2
244598_at	LCP2
244801_at	PSMB7
1552264_a_at	MAPK1
1555613_a_at	ZAP70
1558971_at	THEMIS
1558972_s_at	THEMIS

CELLULAR RESPONSE TO TNF PATHWAY

Probe Set ID	Gene Symbol
AFFX-HUMISGF3A/M97935_5_at	STAT1
AFFX-HUMISGF3A/M97935_MA_at	STAT1
AFFX-HUMISGF3A/M97935_MB_at	STAT1
AFFX-HUMISGF3A/M97935_3_at	STAT1
1405_i_at	CCL5
200039_s_at	PSMB2
200814_at	PSME1
200887_s_at	STAT1
201316_at	PSMA2
201317_s_at	PSMA2
201400_at	PSMB3
201762_s_at	PSME2
202243_s_at	PSMB4
202352_s_at	PSMD12
202643_s_at	TNFAIP3
202644_s_at	TNFAIP3
202659_at	PSMB10
202753_at	PSMD6
203396_at	PSMA4
203508_at	TNFRSF1B
203882_at	IRF9
204279_at	PSMB9
204655_at	CCL5
204780_s_at	FAS
204781_s_at	FAS
205641_s_at	TRADD
205841_at	JAK2
205842_s_at	JAK2
206150_at	CD27
206366_x_at	XCL1
206407_s_at	CCL13
206513_at	AIM2
206641_at	TNFRSF17
206854_s_at	MAP3K7
207339_s_at	LTB
208315_x_at	TRAF3
208805_at	KIAA0391///PSMA6
209040_s_at	PSMB8
209295_at	TNFRSF10B

209341_s_at	IKBKB
209435_s_at	ARHGEF2
209602_s_at	GATA3
209603_at	GATA3
209604_s_at	GATA3
209924_at	CCL18
209969_s_at	STAT1
210538_s_at	BIRC3
210865_at	FASLG
211333_s_at	FASLG
213373_s_at	CASP8
214038_at	CCL8
214228_x_at	TNFRSF4
214567_s_at	XCL1///XCL2
215346_at	CD40
215719_x_at	FAS
221571_at	TRAF3
221903_s_at	CYLD
221905_at	CYLD
222034_at	GNB2L1
222292_at	CD40
32128_at	CCL18
60084_at	CYLD
222868_s_at	IL18BP
223501_at	TNFSF13B
223502_s_at	TNFSF13B
227357_at	TAB3
230499_at	BIRC3
231775_at	TNFRSF10A
244801_at	PSMB7
1555759_a_at	CCL5

T CELL COSTIMULATION PATHWAY

Probe Set ID	Gene Symbol
204890_s_at	LCK
204891_s_at	LCK
205027_s_at	MAP3K8
205868_s_at	PTPN11
206804_at	CD3G
208728_s_at	CDC42
208877_at	PAK2
208935_s_at	LGALS8
209670_at	TRAC
209671_x_at	TRAC
210031_at	CD247
210915_x_at	TRBC1///TRBV19
210972_x_at	TRAC///TRAJ17///TRAV20///TRDV2
211796_s_at	TRBC1///TRBC2///TRBV19///TRBV3-1///TRBV5-4///TRBV6-5///TRBV7-2
211902_x_at	YME1L1
213193_x_at	TRBC1///TRBV19
213539_at	CD3D
213830_at	YME1L1
215536_at	HLA-DQB2
216191_s_at	TRDV3
217143_s_at	TRDC
223501_at	TNFSF13B
223502_s_at	TNFSF13B
227458_at	CD274
236341_at	CTLA4
1555691_a_at	KLRC4-KLRK1///KLRK1
