# Supplementary material for: Paired Rheumatoid Arthritis Synovial Biopsies From Small and Large Joints Show Similar Global Transcriptomic Patterns With Enrichment of Private Specificity TCRB and TCR Signaling Pathways
Source: Front Immunol. 2020 Nov 23;11:593083. doi: 10.3389/fimmu.2020.593083 (PMC7719799; doi:10.3389/fimmu.2020.593083)
Supplement: Supplementary Table 3 — Correlation between TCR signaling gene score, CD3+ histological score and TCRB/RPP30 expression ratio (qPCR). [file DataSheet_3.pdf]

SUPPLEMENTARY TABLE 3
Correlation between TCR signaling gene score, CD3+ histological score and *TCRB/RPP30* expression ratio (qPCR).

	TCR-signaling gene score	CD3+ histological score	*TCRB/RPP30* ratio
TCR-signaling gene score	/	0.6964, *p*=0.0013	0.7576, *p*=0.0001
CD3+ histological score		/	0.6861, *p*=0.0017
TCRB/RPP30 ratio			/

Spearman *r* correlation coefficients and corresponding *p* values
