# Supplementary material for: Paired Rheumatoid Arthritis Synovial Biopsies From Small and Large Joints Show Similar Global Transcriptomic Patterns With Enrichment of Private Specificity TCRB and TCR Signaling Pathways
Source: Front Immunol. 2020 Nov 23;11:593083. doi: 10.3389/fimmu.2020.593083 (PMC7719799; doi:10.3389/fimmu.2020.593083)
Supplement: Supplementary Table 4 — Enriched TCRB sequences in RA synovial biopsies. [file DataSheet_4.pdf]

Supplementary Table 4: Enriched *TCRB* sequences in RA synovial biopsies

TCRB CDR3 sequences	Shared between pair	Patient ID
TRBV_6_TGTGCCAGCAGTTACTCCGAGACCAGGGCCAACGTCCTGACTTTC_TRBJ2-6	Y	1
TRBV_10_TGCGCCAGCAGTGAGTCGTCGACGGTGTCTTACGAGCAGTACTTC_TRBJ2-7	Y	1
TRBV_27_TGTGCCAGCAGTCCTGGCCAGATCGGCTCCTACAATGAGCAGTTCTTC_TRBJ2-1	Y	1
TRBV_12_TGTGCCAGCAGTTTGTACAGGGGAGAGAAACTGTTTTTT_TRBJ1-4	Y	1
TRBV_6_TGTGCCAGCAGTTACCGGGGGCAGAACACTGAAGCTTTCTTT_TRBJ1-1	Y	1
TRBV_6_TGTGCCAGCAGTGACTTCGCGGGCCCCACAGATACGCAGTATTTT_TRBJ2-3	Y	2
TRBV_27_TGTGCCAGCAGTTCGGGCCGACTAGCGGGCTACAATGAGCAGTTCTTC_TRBJ2-1	Y	2
TRBV_5_TGTGCCAGCAGCCCCTGGACAGGGAGCGAGCAGTACTTC_TRBJ2-7	Y	2
TRBV_10_TGCGCCAGCAGTGAGCCGGGACGGAATTCTGAAGCTTTCTTT_TRBJ1-1	Y	2
TRBV_19_TGTGCCAGTAGCCTAAACGGACAGGGGTGGTACTACGAGCAGTACTTC_TRBJ2-7	Y	2
TRBV_24_TGTGCCACCAGTGATTTGCGGAGGGGCGGAGAGACCCAGTACTTC_TRBJ2-5	Y	2
TRBV_29_TGCAGCGTTGAGGACAGGATTTTGGCTCAAGAGACCCAGTACTTC_TRBJ2-5	Y	2
TRBV_19_TGTGCCAGTAGTATGTCCTCCGTGAACACTGAAGCTTTCTTT_TRBJ1-1	Y	2
TRBV_7_TGTGCCAGCAGCTTGTCGGGCCCCGAGGCGAACTATGGCTACACCTTC_TRBJ1-2	Y	2
TRBV_10_TGTGCCATCAGCGACGCCGGGAGCGGTCCCGACAATGAGCAGTTCTTC_TRBJ2-1	Y	2
TRBV_6_TGTGCCAGCAGTTACTCGCAGGGTGTCCCAGATACGCAGTATTTT_TRBJ2-3	Y	2
TRBV_19_TGTGCCAGTAGTATAGATAGGTGGAGCTCCTACAATGAGCAGTTCTTC_TRBJ2-1	Y	3
TRBV_7_TGTGCCAGCAGCGGAAGGCGGGGGCCCCAAGAGACCCAGTACTTC_TRBJ2-5	Y	3
TRBV_6_TGTGCCAGCAGTCCAGACAGCAACGTCCTGACTTTC_TRBJ2-6	Y	4
TRBV_11_TGTGCCAGCAGCTCCGAGGGGACTAGCGTGACAGATACGCAGTATTTT_TRBJ2-3	Y	4
TRBV_12_TGTGCCAGCAGTTTAGGAGGGACTAGCGGGAGATTTGGTAATGAGCAGTTCTTC_TRBJ2-1	Y	4
TRBV_28_TGTGCCAGCAGTTTACGGGGCAGGGCCCGGGTCACTGAAGCTTTCTTT_TRBJ1-1	Y	4
TRBV_5_TGTGCCAGCAGCTTTTCCATCGGGACAGGGATTGAGCAGTTCTTC_TRBJ2-1	Y	4
TRBV_6_TGTGCCAGCAGTGAACAGGCCGGGAGCACAGATACGCAGTATTTT_TRBJ2-3	Y	4
TRBV_6_TGTGCCAGCAGGCCACCGGAGGCGTTGGTAGCTTTCTTT_TRBJ1-1	Y	4
TRBV_7_TGTGCCAGCAGCTTAGTGTCTAGCAACAATGAGCAGTTCTTC_TRBJ2-1	Y	4
TRBV_7_TGTGCCAGCAGCTTAGCCCGGGGGAGCTACGAGCAGTACTTC_TRBJ2-7	Y	4
TRBV_7_TGTGCCAGCAGCGCTAGCGGGAGCACAGATACGCAGTATTTT_TRBJ2-3	Y	4
TRBV_9_TGTGCCAGCAGCGTAGCGGGGGGATCAACCGGGGAGCTGTTTTTT_TRBJ2-2	Y	4
TRBV_3_TGTGCCAGCAGCCAAGCCAACCGGGACAGGGGTTACGAGCAGTACTTC_TRBJ2-7	N	5
TRBV_7_TGTGCCAGCAGTCCCGTAGGAGGGAAAAATTCACCCCTCCACTTT_TRBJ1-6	N	5
TRBV_3_TGTGCCAGCAGCCCTCAATCCAATGAGCAGTTCTTC_TRBJ2-1	N	5
TRBV_28_TGTGCCAGCAGCCATAATCAGCCCCAGCATTTT_TRBJ1-5	Y	5
TRBV_19_TGTGCCACTCTTCCAAACAGAAACTATGGCTACACCTTC_TRBJ1-2	Y	5
TRBV_7_TGTGCCAGAGGGACAGGGGTTAACACCGGGGAGCTGTTTTTT_TRBJ2-2	Y	5
TRBV_7_TGTGCCAGCAGCTTGGGAGGCACTGAAGCTTTCTTT_TRBJ1-1	N	6
TRBV_11_TGTGCCAGCAGCCCACGCGGGAGGAGTCGCGGTCGGAGAATGAGCAGTTCTTC_TRBJ2-1	N	6
TRBV_7_TGTGCCAGCAGCCCCCGACCGACAGGGGTGGGGGCCAACGTCCTGACTTTC_TRBJ2-6	N	6
TRBV_7_TGTGCCAGCAGTCAGGGGGATTTGAACACTGAAGCTTTCTTT_TRBJ1-1	N	6
TRBV_7_TGTGCCAGCAGCCTGGGAGGTACTGAAGCTTTCTTT_TRBJ1-1	N	6
TRBV_19_TGTGCCAGTAGTCTCTGGCGCGGGTCTAGCGGGACCTACAATGAGCAGTTCTTC_TRBJ2-1	N	6
TRBV_20_TGCAGTGCTAGTCTAGCGGGAGGAACAGATACGCAGTATTTT_TRBJ2-3	N	6
TRBV_4_TGCGCCAGCAGCCAAGATGGCGGGGCCGGGGAGCTGTTTTTT_TRBJ2-2	N	6

TRBV_14_TGTGCCAGCAGCCAAGGTCGACTTGGGGGAAACATTCAGTACTTC_TRBJ2-4	N	6
TRBV_4_TGCGCCAGCAGCTACCGACAGGGGTACTCTGGGGCCAACGTCCTGACTTTC_TRBJ2-6	Y	6
TRBV_4_TGCGCCAGCAGCCAAGACCCCAGCATGAACACTGAAGCTTTCTTT_TRBJ1-1	Y	6
TRBV_9_TGTGCCAGCAGCGGGACTTTCGGGACAGGGTTCGGAGATACGCAGTATTTT_TRBJ2-3	Y	6
TRBV_7_TGTGCCAGCAGCCTACGTTCGGAGGATACGCAGTATTTT_TRBJ2-3	N	7
TRBV_28_TGTGCCAGCACCGAAGGACTAGCGGGAGTACAGTTCTACGAGCAGTACTTC_TRBJ2-7	Y	7
TRBV_11_TGTGCCAGCAGCCGGGACAACTACAATGAGCAGTTCTTC_TRBJ2-1	Y	7
TRBV_6_TGTGCCAGCAGTTACACCCCCTGGGCGGGAGGCCCCCACAATGAGCAGTTCTTC_TRBJ2-1	Y	7
TRBV_9_TGTGCCAGCAGCGTAGGCCGGGGCGTTTCCTACAATGAGCAGTTCTTC_TRBJ2-1	Y	7
TRBV_7_TGTGCCAGCAGCTCCGGACAACTTTCCGGTTACGAGCAGTACTTC_TRBJ2-7	Y	7
TRBV_13_TGTGCCAGCAGCTTAGGTACGCGTCAGCAGTACTTC_TRBJ2-7	Y	7
TRBV_9_TGTGCCAGCAGCAAGACCTCCAGGGGAACGAACACCGGGGAGCTGTTTTTT_TRBJ2-2	N	8
TRBV_15_TGTGCCACCAGCAGAGATACTAGCGTGCACACAGATACGCAGTATTTT_TRBJ2-3	N	8
TRBV_28_TGTGCCAGCAGACCAGCGGGGATTAACTACGAGCAGTACTTC_TRBJ2-7	N	8
TRBV_10_TGTGCCATCAGTTTACAGGGAGGCGGCAATCAGCCCCAGCATTTT_TRBJ1-5	N	8
TRBV_5_TGTGCCAGCAGCTTCGTGAGTTCCACAGATACGCAGTATTTT_TRBJ2-3	N	8
TRBV_7_TGTGCCAGCAGCGAGAACAGAGACAATGAGCAGTTCTTC_TRBJ2-1	N	8
TRBV_20_TGCAGTGCTGGCGGTTCTAGCTCCTACGAGCAGTACTTC_TRBJ2-7	N	8
TRBV_6_TGTGCCAGCAGTGCTCTAGCGGGAGGGTCGCAAGAGACCCAGTACTTC_TRBJ2-5	N	8
TRBV_6_TGTGCCAGCAGTTTAAGGGGCAACGAGCAGTTCTTC_TRBJ2-1	N	8
TRBV_5_TGCGCCAGCAGCCCGGCCGGTGGATACAATGAGCAGTTCTTC_TRBJ2-1	N	8
TRBV_28_TGTGCCAGCAATACGCTAGCGGTCTCCTCCTACAATGAGCAGTTCTTC_TRBJ2-1	N	8
TRBV_6_TGTGCCAGCAGTTACAGTACGACGGGAGAGGACGAGCAGTACTTC_TRBJ2-7	N	8
TRBV_5_TGTGCCAGCAGCTTTGGACAGCTTAGCACAGATACGCAGTATTTT_TRBJ2-3	N	8
TRBV_27_TGTGCCAGCAGTTTAGCCGATTGGACAGATACGCAGTATTTT_TRBJ2-3	Y	8
TRBV_7_TGTGCCAGCAGCTCAGACATCGGTGGCTACACCTTC_TRBJ1-2	Y	8
TRBV_12_TGTGCCAGCAGCCACCCGTCGGGACAGGGAAATTCACCCCTCCACTTT_TRBJ1-6	Y	8
TRBV_19_TGTGCCAGTAGTATGGGGCAGGTAGTCAATCAGCCCCAGCATTTT_TRBJ1-5	Y	8
TRBV_5_TGCGCCAGCAGCACAGACAGGGTTGGCCAAGAGACCCAGTACTTC_TRBJ2-5	Y	8
TRBV_3_TGTGCCAGCAGCCAAGATGATCGGGGAGGTCAGCCCCAGCATTTT_TRBJ1-5	Y	8
TRBV_7_TGTGCCAGCAGCTTAGACGGGGGGACTAATGAAAAACTGTTTTTT_TRBJ1-4	Y	8
TRBV_27_TGTGCCAGCAGTGGACTAGCGGGGCGGGATGAGCAGTTCTTC_TRBJ2-1	N	9
TRBV_20_TGCAGTGCTACCGGAGGGTGGCAGCGCAATCAGCCCCAGCATTTT_TRBJ1-5	N	9
TRBV_9_TGTGCCAGCAGCGTAGCGGGGCGGGGGGGCCCAGATACGCAGTATTTT_TRBJ2-3	N	9
TRBV_3_TGTGCCAGCAGCCAAACAGGGTTCGGGGAGCTGTTTTTT_TRBJ2-2	N	9
TRBV_28_TGTGCCAGCAGTCTTTCAGGGTCCTACGAGCAGTACTTC_TRBJ2-7	N	9
TRBV_12_TGTGCCAGCAGTTTAGCCCCCCATTTGGTGAACACTGAAGCTTTCTTT_TRBJ1-1	N	9
TRBV_12_TGTGCCAGCAGCCCGGCGGGGATAAGCACAGATACGCAGTATTTT_TRBJ2-3	N	9
TRBV_6_TGTGCCAGCAGTTCCCCCGTAGTGGGCAATCAGCCCCAGCATTTT_TRBJ1-5	Y	9
TRBV_5_TGCGCCAGCAGCTGGGGACAGGGCGTAGGTGAAAAACTGTTTTTT_TRBJ1-4	Y	9
TRBV_30_TGTGCCTGGAGTGTGGGGACAGGGCTAGCCAAAAACATTCAGTACTTC_TRBJ2-4	Y	9
TRBV_7_TGTGCCAGCAGCTTATGGAATTCCTACGAGCAGTACTTC_TRBJ2-7	Y	9
TRBV_20_TGCAGAGTTTCGGGGAGTATCCACGAGACCCAGTACTTC_TRBJ2-5	N	10
TRBV_7_TGTGCCAGCAGCTCGGGACGGCTTCTGGGTTCACCCCTCCACTTT_TRBJ1-6	N	10
TRBV_6_TGTGCCAGTGGGGACAGAGCCTCACACCATGGCTACACCTTC_TRBJ1-2	N	10
TRBV_19_TGTGCCAGTAGTAAAACTAGCGGTAGTTACAATGAGCAGTTCTTC_TRBJ2-1	Y	10
TRBV_19_TGTGCCAGTAGTTCGACTAGCGGGAGTGTGAATGAGCAGTTCTTC_TRBJ2-1	Y	10
